# Impaired Terminal Differentiation of Hippocampal Granule Neurons and Defective Contextual Memory in *PC3/Tis21* Knockout Mice

**DOI:** 10.1371/journal.pone.0008339

**Published:** 2009-12-17

**Authors:** Stefano Farioli-Vecchioli, Daniele Saraulli, Marco Costanzi, Luca Leonardi, Irene Cinà, Laura Micheli, Michele Nutini, Patrizia Longone, S. Paul Oh, Vincenzo Cestari, Felice Tirone

**Affiliations:** 1 Institute of Neurobiology and Molecular Medicine, Consiglio Nazionale delle Ricerche, Fondazione S.Lucia, Rome, Italy; 2 Institute of Neuroscience, Consiglio Nazionale delle Ricerche, Rome, Italy; 3 LUMSA University, Faculty of Educational Science, Rome, Italy; 4 Molecular Neurobiology Unit, Fondazione S.Lucia, Rome, Italy; 5 Department of Physiology and Functional Genomics, University of Florida, Gainesville, Florida, United States of America; Tokyo Medical and Dental University, Japan

## Abstract

Neurogenesis in the dentate gyrus of the adult hippocampus has been implicated in neural plasticity and memory, but the molecular mechanisms controlling the proliferation and differentiation of newborn neurons and their integration into the synaptic circuitry are still largely unknown. To investigate this issue, we have analyzed the adult hippocampal neurogenesis in a *PC3/Tis21*-null mouse model. *PC3/Tis21* is a transcriptional co-factor endowed with antiproliferative and prodifferentiative properties; indeed, its upregulation in neural progenitors has been shown to induce exit from cell cycle and differentiation. We demonstrate here that the deletion of *PC3/Tis21* causes an increased proliferation of progenitor cells in the adult dentate gyrus and an arrest of their terminal differentiation. In fact, in the *PC3/Tis21*-null hippocampus postmitotic undifferentiated neurons accumulated, while the number of terminally differentiated neurons decreased of 40%. As a result, *PC3/Tis21*-null mice displayed a deficit of contextual memory. Notably, we observed that *PC3/Tis21* can associate to the promoter of *Id3*, an inhibitor of proneural gene activity, and negatively regulates its expression, indicating that *PC3/Tis21* acts upstream of *Id3*. Our results identify *PC3/Tis21* as a gene required in the control of proliferation and terminal differentiation of newborn neurons during adult hippocampal neurogenesis and suggest its involvement in the formation of contextual memories.

## Introduction

*PC3*, also known as *Tis21* or *BTG2* (in rat, mouse and human, respectively; see for review [1], [2]), originally isolated as gene induced by nerve growth factor, negatively controls a cell cycle checkpoint at the G1 to S phase transition in fibroblasts and neuronal cells by direct inhibition of the activity of *cyclin D1* promoter [3], [4], [5]. A number of studies in vivo have shown that *PC3/Tis21* expression is associated with the neurogenic asymmetric division in neural progenitor cells [6], [7], [8], [9], [10]. Moreover, when overexpressed in neural cells, PC3/Tis21 promotes their differentiation [11], [12]. Such pro-differentiative action appears to be consequent not only to inhibition of cell cycle progression but also to a *PC3/Tis21*-dependent activation of proneural genes in neural progenitor cells [12]. *PC3/Tis21* has been shown to regulate and associate with the promoters of *cyclin D1* and *RARβ*, suggesting that it acts as a transcriptional cofactor, being part of transcriptional complexes [13], [14].

The expression of *PC3/Tis21* has also been detected in the dentate gyrus of adult hippocampus, in type-2 progenitor cells as well as in differentiated neurons [15]. Recently, by means of a transgenic mouse over-expressing *PC3/Tis21* in adult hippocampal progenitor cells we have shown that *PC3/Tis21* accelerates their differentiation, without affecting the final number of differentiated neurons. We found that the synaptic plasticity in the dentate gyrus and the performance in different hippocampus-dependent spatial learning and memory tests was severely reduced. This suggested that the time the young neurons spend in different states of neuronal differentiation is critical for their ultimate function in learning and memory [16].

The hippocampus is known to be required in the processing of spatial and contextual memories [17], [18]. A specific role in this context appears to be played by the new neurons continuously generated during adulthood from progenitor cells in the subgranular zone of the dentate gyrus. In fact, adult hippocampal neurogenesis appears to be necessary for hippocampus-dependent learning and memory, as indicated by severe cognitive deficits following ablation of neurogenesis in mice models by toxins, x-ray irradiation or virus-activated pro-drugs [19], [20], [21]. As suggested by several studies, the new adult neurons might be used for storing new memories, thus protecting older memories from interference, or for encoding temporally proximal events [22], [23], [24], [25]. Nonetheless, the ablation of new neurons did not affect hippocampus-dependent tasks in a number of studies [26], [27], raising questions about the precise role of new neurons in learning and memory. Moreover, the molecular mechanisms that coordinately govern in newborn neurons the processes of proliferation, differentiation and integration into the memory circuits are still incompletely understood.

Thus, in this study we wished to assess whether *PC3/Tis21* exerted a physiological role in the control of neurogenesis. In fact, during brain development *PC3/Tis21* is expressed in the proliferating neuroblasts of the ventricular zone of the neural tube, and to a lower extent in the differentiating neuroblasts of the mantle zone; postnatally it is expressed in cerebellar precursors mainly in the proliferating regions of the neuropithelium (i.e., in the external granular layer), and in the hippocampus in proliferating and differentiating progenitor cells [6], [12], [16], [15]. Hence, given that *PC3/Tis21* appears to be a pan-neural gene that marks neural progenitors and induces their differentiation, its genetic ablation is expected to interfere with the ongoing neurogenesis. Indeed, we found that the terminal differentiation of adult-generated hippocampal granule neurons is specifically dependent on *PC3/Tis21* expression, being impaired in mice ablated of *PC3/Tis21*.

Such impairment was accompanied by a deficit of contextual memory in fear-related tests, suggesting that terminal differentiation of new neurons can be critical for associative memory.

## Methods

### *PC3/Tis21*-Null Mice and Genotyping

The *PC3/Tis21* knockout mice had been generated previously, as described [28]. Mutant mice were of the C57BL/6 (B6) strain and had a replacement of the entire exon II of the Tis21 gene (referred to as *PC3/Tis21* throughout this paper), which spans from aa 49 to 158 and includes the boxes A and B, with the neomycin resistance cassette. Genotypization of mice was routinely performed by PCR, using genomic DNA from tail tips. Two pairs of primers were used to identify mice carrying the different alleles *PC3/Tis21* ^−/−^, *PC3/Tis21* ^+/−^ or *PC3/Tis21* ^+/+^, one complementary to the neo cassette and another complementary to the targeted exon II, and were amplified together in the PCR reaction to obtain different patterns of amplification specific for each of the three combinations of alleles: neo(+) 5′-AGGCTATTCGGCTATGACTG-3′, neo(−) 5′-GTCAAGAAGGCGATAGAAGG-3′ (712 bp amplification); exII(+) 5′-CATCCAAGGTTCTGGCTATC-3′, exII(−) 5′-GCCATCACTAGTTCTTCGAG3′ (270 bp amplification, i.e., the whole exon II).

Mice were maintained under standard specific-pathogen-free conditions, and underwent behavioral testing during the second half of the light period (between 2:00 and 5:00 p.m.) in sound-insulated rooms. All animal procedures were completed in accordance with the Istituto Superiore di Sanita' (Italian Ministry of Health) and current European (directive 86/609/ECC) Ethical Committee guidelines.

### BrdU Treatment of Mice and Sample Preparation for Immunohistochemistry

Postnatal day 60 (P60) *PC3/Tis21* ^−/−^ and *PC3/Tis21* ^+/+^ mice were analyzed after treatment with five daily injections of bromodeoxyuridine (BrdU; 95 mg/kg i.p.) to detect dividing adult neurons in dentate gyrus, in the subventricular zone, and in the olfactory bulb [16]. To detect new progenitor cells entering in S-phase in the dentate gyrus, P60 mice were analyzed after 2 hours treatment with BrdU, while P14 mice after one hour treatment, according to previous protocols [29], [30]. New progenitor cells entering in S-phase in the subventricular zone were detected after 2 hour treatement with BrdU, following previous protocols [31]. Brains were collected after transcardiac perfusion with 4% paraformaldehyde (PFA) in PBS –DEPC and kept overnight in PFA. Afterwards, brains were equilibrated in sucrose 30% and cryopreserved at −80°C.

### Quantification of Cell Numbers and Volumes

Stereological analysis of the number of cells was performed on one-in-six series of 40-µm freefloating coronal sections (240 µm apart), which were analyzed by confocal microscopy to count cells expressing the indicated marker throughout the whole rostro-caudal extent of the dentate gyrus, of the subventricular zone and of the olfactory bulb. To obtain the total estimated number of cells within the dentate gyrus, positive for each of the indicated markers, the average number of positive cells per section was multiplied by the total number of 40-µm sections comprising the entire dentate gyrus, as described [32], [33], [34], [16]. Three animals per group were analyzed. The same procedure was used to measure cell numbers in the subventricular zone and in the olfactory bulb.

Stereological analysis of the volumes and of the absolute number of granule cells in the dentate gyrus was performed analyzing every eighth section in a series of 40-µm coronal sections, thus spaced 320 µm. Total cell number was obtained according to the optical disector principle, by systematic sampling of counting frames of 15-µm side in each section. Nuclei (stained with Hoechst 33258) that appeared in the different focal planes of the frame were included in the count, while nuclei in the uppermost focal plane of each section and intersecting the exclusion boundaries of the counting frame were excluded, as defined by the optical disector principle [35]. Total cell number (N) was calculated using the formula N = Nv x Vref, where Nv is the average cell number per disector volume (corresponding to 15×15×40 µm3) and Vref (reference volume) is the total volume of the dentate gyrus. The reference volume was obtained multiplying the sum of the traced areas of the dentate gyrus or hippocampus by the 320 µm distance between sections analyzed [35]. Labeled cells and areas were measured by computer-assisted analysis using the I.A.S. software (Delta Systems, Rome, Italy).

### Immunohistochemistry and BrdU Labeling

Immunohistochemistry was performed on serial freefloating sections cut at 40-µm thickness for hippocampus as well as for the subventricular zone and olfactory bulb, at -25°C in a cryostat from brains embedded in Tissue-Tek OCT (Sakura, Torrence, CA). Sections were then stained for multiple labeling using fluorescent methods. BrdU incorporation was detected following pretreatment of sections to denature the DNA, with 2 N HCl 45 min at 37°C and then with 0.1 M sodium borate buffer pH 8.5 for 10 min. Afterwards, sections were incubated with a rat monoclonal antibody against BrdU (Serotech, Raleigh, NC; MCA2060; 1∶150) together with other primary antibodies, as indicated. Namely, mouse monoclonal antibodies raised against nestin (Chemicon International, Temecula, CA; MAB353; 1∶150), NeuN (Chemicon International; MAB377; 1∶100) and PH3 (Cell Signaling Technology, Danvers, MA; 9706; 1∶100), or goat polyclonal antibodies against Doublecortin (DCX; Santa Cruz Biotechnology, Santa Cruz, CA; SC-8066; 1∶200), Calretinin (Chemicon International; AB1550; 1∶400), Sox2 (Santa Cruz Biotechnology; Sc17320, 1∶400), NeuroD1 (R&D Systems, Minneapolis, MN; AF2746; 1∶100), Calbindin (Santa Cruz Biotechnology; Sc-7691, 1∶100), or rabbit polyclonal antibodies against Glial fibrillary acidic protein (GFAP; Promega Corporation, Madison, WI; G560A; 1∶150), Calretinin (Swant, Bellinzona, Switzerland; 7699/4; 1∶500), Caspase-3 (Cell Signaling Technology, Danvers, MA; 9661; 1∶100) or c-fos (Chemicon International; Ab-5 PC38T; 1∶500), Calbindin (Chemicon International; AB1778, 1∶200), Id3 (Santa Cruz Biotechnology; Sc-490, 1∶400), Tbr2 (Chemicon International; AB9618, 1∶100), or the rabbit monoclonal antibody against Ki67 (LabVision Corporation, Fremont, CA; SP6; 1∶100). Secondary antibodies used to visualize the antigen were either donkey anti-rat TRITC (tetramethylrhodamine isothiocyanate)-conjugated (Jackson ImmunoResearch, West Grove, PA, USA; BrdU), or donkey anti-rabbit TRITC-conjugated (Jackson ImmunoResearch; Calretinin, Caspase-3), donkey antimouse or donkey anti-rabbit Alexa 647 (Invitrogen, San Diego, CA; nestin and NeuN or c-fos, respectively), donkey anti-rabbit Cy2-conjugated (Jackson ImmunoResearch; Ki67, GFAP), or donkey anti-goat Cy2-conjugated (Jackson ImmunoResearch; Calretinin, Sox2, DCX, NeuroD1).

Images of the immunostained sections were obtained by laser scanning confocal microscopy using a TCS SP5 microscope (Leica Microsystem). Analyses were performed in sequential scanning mode to rule out cross-bleeding between channels.

### Cell Culture

PC12 cells expressed conditionally *PC3* (rat sequence), under control of the tet-off system [36]. They were generated by transfecting a PC12 cell clone carrying CMV-tTA-neo (tetracycline-regulated transactivator, driven by the CMV promoter; Clontech Laboratories, Mountain View, CA) with the construct TRE-*PC3* (pUHD10-3-PC3; tetracycline responsive element), previously generated by us [12], and with the vector pBABE-puro (1/10 molar ratio) to allow selection of clones by puromycin. One PC12 cell clone carrying both constructs CMV-tTA and TRE-*PC3* was chosen, named PC12/tet-off-PC3; the tTA protein produced constitutively by CMV-tTA activates TRE-*PC3* in the absence of doxycycline (tetracycline analog). The PC12/tet-off-*PC3* cell clone was routinely grown in Dulbecco's modified Eagle's medium (DMEM) containing 5% fetal calf serum and 10% horse serum (HyClone, Logan, Utah) with doxycycline (1 µg/ml), and also G418 (100 µg/ml) and puromycin (2 µg/ml) to maintain the selection, in a humidified atmosphere of 10% CO_2_ at 37°C.

### Chromatin Immunoprecipitation (ChIP)

Regarding ChIP assays, purification of nuclei, preparation of chromatin by micrococcal nuclease digestion (to obtain fragments of predominantly one to five nucleosomes) and immunoprecipitation with anti-PC3/Tis21 antibody, were performed as described previously, with minor modifications [37]. The anti-PC3/Tis21 antibody used for immunoprecipitations was A3H [4], while normal rabbit serum was used as control.

Approximately 4×10^7^ tet-off-*PC3* PC12 cells, conditionally expressing *PC3* and harvested at the stages of differentiation indicated, were used for each immunoprecipitation. The immunoprecipitated DNA and 1/200 dilution of the input DNA were analyzed in triplicate samples by real-time PCR using SYBR Green PCR Master Mix (Applied Biosystems, Foster City, CA, USA) and a 7900HT System (Applied Biosystems). The immunoprecipitated DNA promoter was quantified using the 2^-Ct^ formula and was calculated as input percentage (ratio of the average value of the DNA detected in immunoprecipitated samples to the average value of the DNA present in input lysates; as described by Heard et al., [38]). For each cell treatment we calculated in parallel the input percentages of DNA immunoprecipitated by immune and by normal rabbit serum. The amount of DNA promoter immunoprecipitated was finally expressed as fold enrichment, meant as ratio of input percentage of DNA immunoprecipitated by A3H antibody to input percentage of DNA immunoprecipitated by normal rabbit serum.

PCR primers used to amplify: i) the *Id3* promoter (region of 700 nt before transcription start): 5′-AAGATAATTCCTGACGCCAGTGAG-3′, 5′-AATTAGTGCCGCCTTG TTCCC-3′; ii) the MCK promoter (region 340 nt before transcription start): 5′-GGCTGAGGGCAGGCTGTAAC-3′, 5′-GGGTCAG TAATACTCTGGGTGTCC-3′. The sequence of primers amplifying the promoters of other genes analyzed (*NeuroD1*, *Hes1*, *Mash1*, *Ngn1*, *Msx1*, *Id1*) are available upon request.

### RNA Extraction and Real Time-PCR

Total cellular RNA was extracted from tet-off-*PC3* PC12 cells and reverse-transcribed as described previously [5]. Real-time PCR was performed with a 7900HT System (Applied Biosystems) using SYBR GreenI dye chemistry in duplicate samples. Relative quantification was performed by the comparative cycle threshold method [39]. The expression values of *PC3* and *Id3* in PC12 cells and in the dentate gyrus were normalized to TATA-binding protein RNA, which was used as endogenous control.

Specific real-time RT-PCR primers used to amplify were as follows: *PC3* (rat sequence): 5′-GCGAGCAGAGACTCAAGGTT-3′, 5′- CCAGTGGTGTTTGTAAT GATCG-3′; *Id3*: 5′-AGGAGTTTTTGCCACTGACC-3′, 5′-CTCATCCATGC CCCTCAG-3′; TATA-binding protein: 5′-GCTACTTGGGCGGCACTG-3′, 5′-ACCAACAATCACCAGCAG CAG-3′

### In Situ Hybridization

Preparation of sections and hybridization were performed as reported previously [12]. Antisense riboprobe detecting *Id3* mRNAs was synthesized by SP6 polymerase from the 3′ UTR region of mouse *Id3* cDNA [40], a 335 bp long sequence part of the third exon of *Id3* and devoid of any cross-homology to Id1 and Id2 sequences, previously cloned by us into the pcDNA3 vector, and restricted by HindIII. The pcDNA3-*Id3* construct was obtained by cloning the 3′ UTR *Id3* cDNA region - amplified using genomic mouse DNA as template - into the EcoRI5′-HindIII3′ sites of the pcDNA3 vector, and was checked by sequencing. Riboprobes were labeled with digoxigenin-UTP (Transcription kit; Roche Products), following the protocol of the manufacturer. No signal was detected by sense probes.

### Behavioral Tests

Male mice (2 months of age; 12 per group of each genotype) were used for behavioral evaluation. The Morris water maze [41] was carried out in a circular swimming pool of 1.3 m in diameter, filled with opaque water at the temperature of 25±1°C and located in a room containing prominent extra-maze cues. A hidden 15-cm-diameter platform was used. The training consisted of 24 trials (4 trials per day, lasting a maximum of 60 sec, with an intertrial interval of 30 min), with the platform left in the same position. A probe test (60 sec) was carried out 24 h after the last trial by removing the platform from the pool. Mice behavior was evaluated by EthoVision software (Noldus Information Technology, Wageningen, NL). Contextual fear conditioning [42], was carried out in a conditioning chamber (26×22×18 cm) made of transparent Plexiglas with a grid metal floor, located in a sound-insulated box lighted by a white tensor lamp (60 W). After an acclimatizing period lasting 180 sec, a foot-shock was delivered (unconditioned stimulus, US; 0.7 mA, 2 sec). Mice were left in the conditioning chamber for a further period of 60 sec and then returned into their home cage. Contextual test (5 min) was performed 24 h after training, in the same chamber. Freezing was defined as the complete absence of motion, except for respiratory movements. Step-through inhibitory avoidance (passive avoidance) was tested in an apparatus previously described [43]. A straight alley was divided into two compartments, one 7.5 cm long and the other 14 cm long. The floor was 2.5 cm wide and the top 10 cm wide. The smaller compartment was made of white Plexiglas. The larger one was made of black Plexiglas and was equipped with a removable cover of the same material to allow the compartment to be in darkness. The two compartments were separated by a sliding door. A tensor lamp (60 W, positioned 80 cm above the apparatus) illuminated the small compartment. The floor of the larger compartment consisted of two oblique stainless-steel plates separated by 0.5 cm at the bottom and 10 cm at the top, through which scrambled constant current could be delivered. The shape of the electrified floor ensured that the mouse made contact with both plates simultaneously in order to receive the shock. On the training day, each mouse was placed in the light compartment, facing away from the dark compartment. When the mouse turned around, the door leading to the dark compartment was opened. When the mouse had stepped with all four paws into the dark side, the door was closed, the step-through latency was recorded, and two foot shocks (0.4 mA, 50 Hz, 2s, 5s ISI) were delivered. Twenty-four hours after training mice were tested in the same apparatus with a similar procedure to that of training, except that no foot shocks were administrated. A maximum step-through latency of 240s was allowed in the test sessions.

### Id3 Promoter Activity

Tet-off-*PC3* PC12 cell cultures expressing *PC3* or not, seeded in 35-mm polylysine-coated dishes containing 5×10^5^ cells, were transfected the following day with the pGL3-*Id3*-prom/-1592 reporter construct using the Lipofectamine reagent, and harvested after 48 hours, being treated in parallel with NGF for the indicated times. Luciferase assays were performed by the Luciferase assay system (Promega) according to the manufacturer's instructions as previously described [12]. Luciferase activities were measured in luciferase units per microgram of protein normalized to the activity of the coreporter pRSV-β-galactosidase present in each extract, as a measure of the efficiency of transfection.

The pGL3-*Id3*-prom/-1592 construct was obtained by cloning in the 5′SacI-3′BglI site of pGL3-basic the PCR-amplified region of the *Id3* promoter (1592 nt before transcrition start), using genomic mouse DNA as template. The construct was checked by sequencing.

### Laser Capture Microdissection and RNA Extraction

One-in-ten series of 30-µm freefloating coronal sections (300 µm apart) comprising the entire hippocampus, obtained from 4% PFA- fixed brains, following dehydration in 100% ethanol and re-hydration in descending alcohols, were incubated in 0.02% Cresyl Violet, sampled on Rnase-free membrane mounted metal frame slides (Molecular Machines & Industries GmbH, Eching, Germany) and dried in a fume hood (1 h) at room temperature, in order to be processed by laser capture microdissection according to a described protocol [44]. Histological slides were examined with an inverted Nikon microscope (Nikon Eclipse TE2000-S) coupled with a UV laser (337 nm) unit and controlling software (SL Microtest GmbH, Jena, Germany) and a CCD camera (Sony, Japan). The dentate gyrus was visualized at 4-20X magnifications and the laser path was directed to the Nissl-stained granule cell layer outline in all the processed sections. The dissection of an equal number of bilateral areas from both wild type and knockout mice was checked by examination of the adhesive lid at the end of every laser capture microdissection session. Tubes were stored at −80°C until RNA extraction.

The Absolutely RNA FFPE kit (Agilent Technologies-Stratagene, CA, USA) was used to isolate RNA from laser capture microdissection samples, following manufacturer's protocol for formalin fixed tissues, excluding any deparaffinizing procedure. The eluted, DNase treated RNA was further precipitated by adding 1 µg/µl glycogen and finally dissolved in 10 µl of DEPC water.

### Statistical Analysis

One-way ANOVA was used to analyze the levels of freezing in the contextual fear conditioning and in the inhibitory avoidance. Morris water maze results were analyzed by two-way ANOVA. Student's t-test was used to analyze number of neurons and hippocampal volumes mRNA levels and Id3 promoter activity.

## Results

### The Development of Adult Hippocampal Granule Precursors Is Arrested before Terminal Differentiation in the Absence of *PC3/Tis21*

We sought in first place to assess whether the development of hippocampal progenitor cells is dependent on *PC3/Tis21* expression. To this end, we used a loss-of-function approach, analyzing in mice ablated of *PC3/Tis21* the maturation of progenitor cells of the dentate gyrus, in the adult hippocampus (P60), or in the developing hippocampus (at P14), when the population of differentiating new progenitor cells is massive [45].

Thus, mice with a null mutation of *PC3/Tis21*, generated by deletion of the second exon that carries the homology boxes A and B, i.e., the active domains of the protein [28], [1], [2], were treated at P55 with five daily injections of BrdU, and immediately after the different cell populations of the dentate gyrus were analyzed. Such BrdU treatment allows to detect new progenitors and neurons 1- to 5-day-old. The process of adult hippocampal neurogenesis from putative neural stem cells to post-mitotic granule neurons has been divided in six developmental stages [46]. The cell populations of the dentate gyrus are believed to originate from the putative neural stem cells with radial glial-like morphology identified by the expression of GFAP in their processes [47], expressing also nestin or Sox2 and defined as type-1 cells [48], [49], [50], [51].

**Figure 1:**
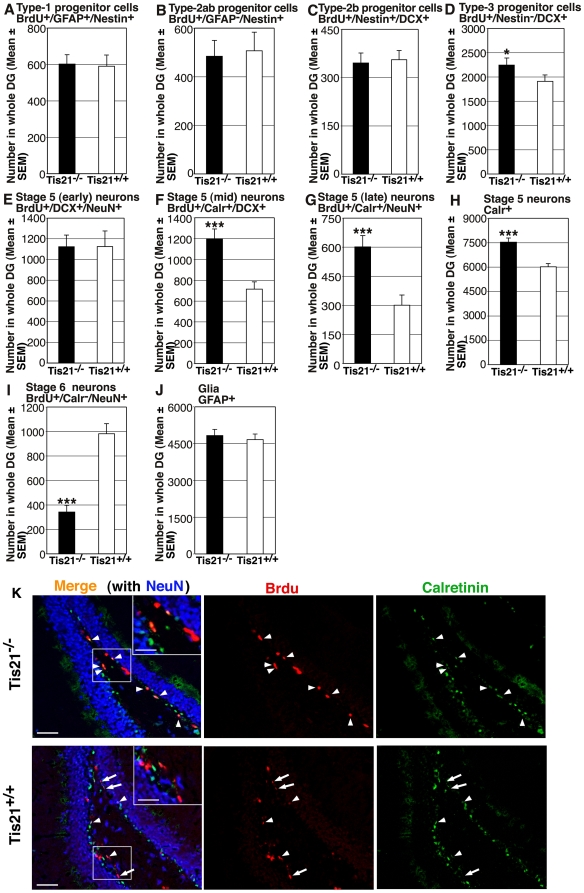
Ablation of *PC3/Tis21* impairs terminal differentiation of 1- to 5-day-old adult-generated dentate gyrus progenitor cells. New stem and progenitor cells (**A–D**) and post-mitotic neurons (**E–I**) of 1 to 5 days of age, as detected by incorporation of BrdU after five daily injections in P60 *PC3/Tis21* ^+/+^ and *PC3/Tis21* ^−/−^ mice (referred to as Tis21 throughout the figures), were analyzed by the expression of specific markers through multiple-labeling confocal microscopy. (**A**) New stem cells (type-1; BrdU/GFAP/nestin-positive) and (**B**) type-2ab (BrdU/nestin-positive and GFAP-negative) or (**C**) type-2b (BrdU/nestin/DCX-positive) progenitor cells did not present significant changes of number in *PC3/Tis21*-null mice. (**D**) Type-3 progenitor cells (BrdU/DCX-positive nestin-negative) increased significantly, of about 17%. (**E**) New stage 5 post-mitotic neurons, derived from progenitor cells and identified by NeuN, did not show evident changes at the onset of differentiation (early stage 5, BrdU/DCX/NeuN-positive), while (**F**) a significant increase of about 2-fold was observed in neurons at a further stage characterized by expression of Calretinin, i.e., in BrdU/Calr/DCX-positive neurons, and (**G**) in BrdU/Calr/NeuN-positive neurons. (**H**) Also the whole population of Calretinin-positive neurons of all ages, was significantly increased in *PC3/Tis21*-null mice (Calr-positive neurons, 25% increase). (**I**) In contrast, the terminally differentiated stage 6 neurons (BrdU/NeuN-positive and Calr-negative) decreased sharply (65% decrease). (**J**) Moreover, glial cells, identified as GFAP-positive, did not change in number, indicating that gliogenesis was not affected. The strong increase of stage 5 neurons, in parallel with the strong decrease of stage 6 neurons, suggests that Calretinin-positive neurons accumulate, being unable to attain terminal differentiation at stage 6. The cell numbers in dentate gyrus, shown in (**A–J**), were measured as described in Materials and Methods and are represented as mean ± SEM of the analysis of three animals per group. ***, *p*<0.001 vs. *PC3/Tis21* ^+/+^ dentate gyrus; Student's t test. (**K**) Representative confocal images showing 1– to 5-day-old stage 5 dentate gyrus neurons positive to BrdU/Calr/NeuN (in orange in the merged image, indicated by arrowheads; single labeling is red, green, blue, respectively), which increase conspicuously in *PC3/Tis21*-null mice, and of stage 6 new neurons positive to BrdU/NeuN and negative to Calretinin (in purple the merged image, indicated by arrows), which in contrast decrease. Insets show Calretinin-positive and -negative cells at higher magnification. Scale bar, 50 µm; 27 µm in the inset.

In *PC3/Tis21*-null mice no difference, with respect to control mice, was observed in number or morphology of type-1 cells (identified as BrdU^+^/GFAP^+^/nestin^+^; Figure 1A), or of the whole type-2a/type-2b population (considered as transiently amplifying progenitor cells derived from type-1 cells, identified as BrdU^+^/GFAP^−^/nestin^+^; Figure 1B), or of type2-b cells, expressing the immature neuronal marker DCX [48], [49]; identified as BrdU^+^/nestin^+^/DCX^+^, Figure 1C). Type-3 progenitor cells, that cease to express nestin but still express DCX [52], significantly increased in number in *PC3/Tis21*-null mice (BrdU^+^/nestin^−^/DCX^+^; about 17% increase; Figure 1D).

As maturation proceeds, the dentate gyrus progenitor cells become post-mitotic, attaining the stage 5 [46], indicated by the expression of the mature neuronal marker NeuN, which coexists initially with DCX and subsequently with Calretinin [53], [54]. Analyzing 1- to 5-day-old neurons we observed that, while those at the beginning of stage 5 did not change in number in *PC3/Tis21*-null mice (BrdU^+^/DCX^+^/NeuN^+^; Figure 1E), a striking two-fold increase was detectable in Calretinin-expressing neurons, either in the less mature that still co-express DCX (BrdU^+^/Calr^+^/DCX^+^; *p* = 0.0003; Figure 1F) or in the whole population of Calretinin-positive neurons co-expressing NeuN (BrdU^+^/Calr^+^/NeuN^+^; *p* = 0.0003; Figure 1G). Consistently, the total population of Calretinin-positive neurons, irrespective of their age, significantly increased (Calr^+^; *p* = 0.00001; Figure 1H). In constrast, we observed a sharp decrease of terminally differentiated neurons, i.e., of neurons expressing NeuN without Calretinin, (stage 6, BrdU^+^/Calr^−^/NeuN^+^; about 65% decrease. *p* = 0.000001; Figure 1I). Representative images of BrdU^+^/Calr^+^/NeuN^+^ and BrdU^+^/Calr^−^/NeuN^+^ neurons are shown in Figure 1K.

As a whole, this indicates that the development of the dentate gyrus neurons undergoes an arrest at the the end of stage 5, so that only few neurons attain terminal differentiation and stage 6. Consistently, we observed in *PC3/Tis21*-null mice a significant decrease of hippocampal granule neurons expressing NeuroD1, which is required for their terminal differentiation ([55]; total NeuroD1^+^ neurons: about 15% decrease, *p* = 0.0011, Figure S1A; BrdU^+^/NeuroD1^+^ neurons: about 22% decrease, *p* = 0.020, Figure S1B; BrdU^+^/NeuroD1^+^/NeuN^+^ neurons: about 35% decrease, *p* = 0.0027, Figure S1C; a representative image is shown in Figure S1D). No difference was observed in glia, identified as GFAP^+^ cells (Figure 1J), indicating that the impairment of neuron differentiation did not result in altered gliogenesis.

As a next step, we checked whether the impairment of differentiation was detectable also in older neurons, of 28 days of age. Thus, we analyzed the hippocampi of P60 mice, four weeks after treatment with five daily injections of BrdU.

**Figure 2:**
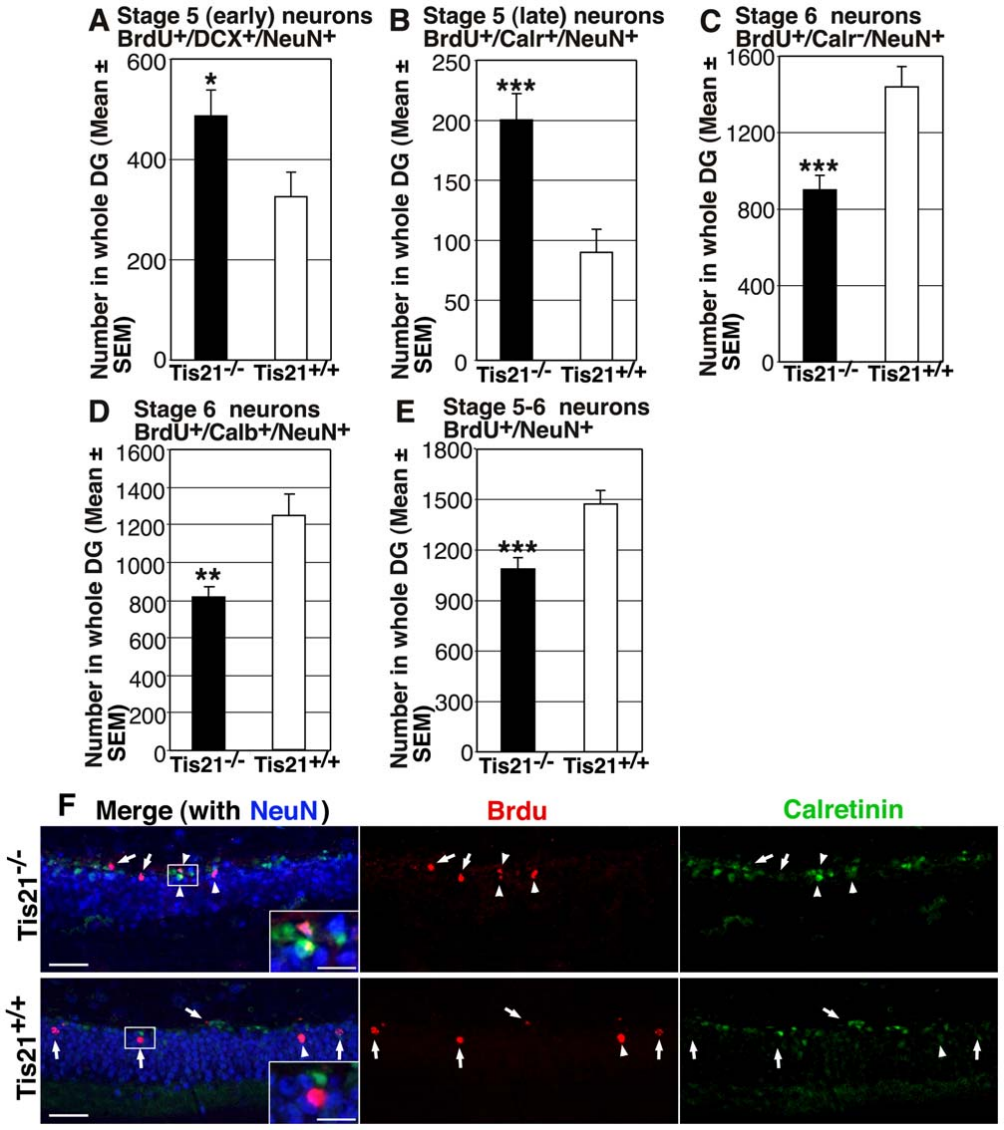
Ablation of *PC3/Tis21* impairs terminal differentiation of 28-day-old adult-generated dentate gyrus progenitor cells. (**A, B**) Neurons of about 28 days of age, as defined by BrdU birthdating, expressing either (**A**) the immature marker DCX (BrdU/DCX/NeuN-positive), or (**B**) Calretinin - that labels neurons not yet terminally differentiated - (BrdU/Calr/NeuN-positive), were significantly increased in *PC3/Tis21*-null mice (50% and 2.2 fold increase, respectively). (**C**, **D**) In contrast, terminally differentiated 28 day-old neurons identified either as (**C**) BrdU/NeuN-positive and Calr-negative or as (**D**) BrdU/Calbindin/NeuN-positive cells, decreased sharply (about 38% and 35%, respectively). (**E**) The population of NeuN-positive neurons, analyzed as a whole, also decreased significantly, albeit to a lower extent (27%). Thus, the impairment of terminal differentiation observed in 1- to 5-day-old neurons (Figure 1) was still present also in older neurons of 4 weeks. Cell numbers are represented as mean ± SEM of the analysis of three animals per group. *, *p*<0.05 or **, *p*<0.01 or ***, *p*<0.001 vs. *PC3/Tis21* ^+/+^ dentate gyrus; Student's t test. (**F**) Representative confocal images showing 4-week-old stage 5 dentate gyrus neurons positive to BrdU/Calretinin/NeuN, which increase in *PC3/Tis21*-null mice (in green or pink in the merged image, indicated by arrowheads; single labeling is red, green, blue, respectively), and of stage 6 new neurons positive to BrdU/NeuN and negative to Calretinin (in purple, indicated by arrows), whose number decreases. It is also detectable an increase of the whole population of Calretinin-positive cells in *PC3/Tis21*-null mice (compare the right panels). Insets show single Calretinin-positive or -negative cells. Scale bar, 45 µm; 15 µm in the inset.

In the dentate gyrus of mutant mice a greater number of immature neurons was detected, not only co-expressing Calretinin with NeuN, but also co-expressing DCX with NeuN (BrdU^+^/DCX^+^/NeuN^+^; 1.5-fold increase, *p* = 0.04; Figure 2A) (BrdU^+^/Calr^+^/NeuN^+^; 2.2-fold increase, *p* = 0.0004; Figure 2B). In contrast, we observed a sharp decrease of the number of 28-day-old terminally differentiated neurons, identified either by expression of NeuN without Calretinin (about 38% decrease; BrdU^+^/Calr^−^/NeuN^+^; *p* = 0.0001; Figure 2C), or by expression of NeuN with the mature neuron marker Calbindin ([54]; BrdU^+^/Calb^+^/NeuN^+^; *p* = 0.001; Figure 2D). Also the whole population of new neurons of 28 days of age, identified as BrdU^+^/NeuN^+^ cells, decreased in number in *PC3/Tis21* ^−/−^ mice (27% decrease, *p* = 0.03; Figure 2E). Representative images of BrdU^+^/Calr^+^/NeuN^+^ and BrdU^+^/Calr^−^/NeuN^+^ neurons are shown in Figure 2F.

Altogether, the neurons of 4 weeks of age in *PC3/Tis21*-null hippocampi showed a delay in attaining terminal differentiation, as observed at 1–5 days of age, indicated by the increase of the population of neurons expressing the immature markers Calretinin and also DCX, in concomitance with a decrease of the terminally differentiated Calr^−^/NeuN^+^ or Calb^+^/NeuN^+^ neurons.

### In the Absence of *PC3/Tis21* Dentate Gyrus Progenitor Cells Show Enhanced Proliferation and Shortened Duration of G1-Phase

**Figure 3:**
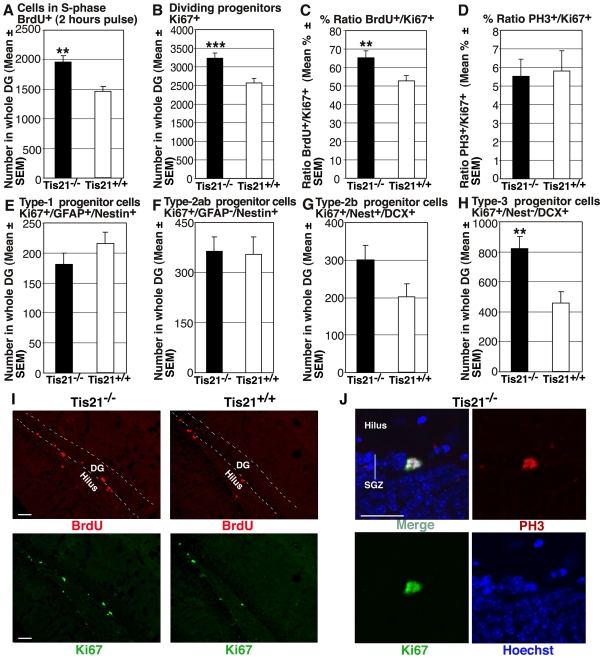
Dentate gyrus progenitor cells proliferate more in mice ablated of *PC3/Tis21*. (**A**) In P60 *PC3/Tis21*-null mice the total number of progenitor cells in the dentate gyrus entering in S-phase, identified by a short pulse of BrdU, increased significantly, of about 30%. (**B**) A similar, significant increase occurred also for the total number of dividing progenitors in the dentate gyrus, measured as Ki67-positive cells, and (**C**) for the ratio between cells in S-phase (BrdU^+^) and all dividing progenitors (Ki67^+^). This ratio, with a net increase of progenitor cells entering in S-phase with respect to the number of dividing cells, indicates that the length of cell cycle (G1 phase) was shorter in *PC3/Tis21*-null mice. (**D**) Consistently, no difference was observed in the ratio between cells in mitosis (PH3^+^) and all dividing progenitors (Ki67^+^). An analysis of the types of dividing progenitor cells types indicates that while the number of (**E**) type-1 (Ki67/GFAP/ nestin-positive) and (**F**) type-2ab (Ki67/nestin-positive and GFAP-negative) did not change significantly, the subpopulation (**G**) type-2b (Ki67/nestin/DCX-positive) and also (**H**) type-3 (Ki67/DCX-positive and nestin-negative) increased, in the latter case significantly, of about 50% and 80%, respectively. These results are consistent with a model where *PC3/Tis21* physiologically inhibits proliferation and favors differentiation. Cell numbers are represented as mean ± SEM of the analysis of three animals per group. **, *p*<0.01 or ***, *p*<0.001 vs. *PC3/Tis21* ^+/+^ dentate gyrus; Student's t test. (**I**) Representative confocal images showing dentate gyrus progenitors either entering in S-phase or dividing, labeled by a 2 hours BrdU pulse (in red), or by Ki67 (in green), respectively. In both cases, their number increases in *PC3/Tis21*-null mice, as compared to WT mice. Scale bar, 50 µm. (**J**) A progenitor cell positive to phospho-histone H3, marker of the G2/M cell cycle phase, in dentate gyrus of *PC3/Tis21*-null mice. PH3, Ki67 and nuclei (labeled by Hoechst 33258) are in red, green and blue, respectively. SGZ, subgranular zone. Scale bar, 30 µm.

The progenitor cells in S-phase, identified by incorporation of a short BrdU pulse of 2 hours [29], [30], as well as the total number of dividing progenitor cells, identified by the proliferation marker Ki67 [56], increased significantly in mutant mice (BrdU^+^, 33% increase, *p* = 0.001, Figure 3A; Ki67^+^, 25% increase, *p* = 0.0009, Figure 3B). Representative images of BrdU^+^ and Ki67^+^ cells are shown in Figure 3I. This indicated that the absence of *PC3/Tis21* led to an increase of proliferation of neural progenitor cells. Furthermore, the fraction of progenitor cells in S-phase (BrdU^+^) among those actively dividing (Ki67^+^) increased significantly in mutant mice (BrdU^+^ vs Ki67^+^ ratio, 23% increase, *p* = 0.008, Figure 3C); this ratio is inversely proportional to the length of the cell cycle [57], [58] and indicated that the G1 phase of dentate gyrus progenitor cells was shortened in mutant mice. In contrast, the fraction of progenitor cells in G2/M-phase (positive to the mitotic marker anti-phospho-histone H3 [PH3]; [59]) among those actively dividing (Ki67^+^), did not differ in mutant and WT mice, confirming that the decrease of cell cycle length concerned the G1 phase (PH3^+^ vs Ki67^+^ ratio, *p* = 0.84, Figures 3D and 3J).

A closer analysis of the proliferative activity of the different progenitor cell populations indicated that, while the number of dividing type-1 progenitor cells (Ki67^+^/GFAP^+^/nestin^+^, Figure 3E) and of the whole type-2ab population (Ki67^+^/GFAP^−^/nestin^+^, Figure 3F) did not increase, the number of type-2b and type-3 increased 50% (Ki67^+^/nestin^+^/DCX^+^, *p* = N.S., Figure 3G) and 80%, respectively (Ki67^+^/nestin^−^/DCX^+^, *p* = 0.003, Figure 3H). Similar results, concerning type-2a/type-2b progenitor cells, were also obtained using Tbr2 as marker, which labels quite selectively this population [60], (Figure S2A-D).

In conclusion, we observed that in *PC3/Tis21*-null mice - in parallel with an accumulation of dentate gyrus neurons not terminally differentiated (stage 5, up to 4-week-old) and with a reduction of terminally differentiated neurons (stage 6) - the type-3 progenitor cells, that originate stage 5 neurons, showed an increase of proliferation and number. This indicates a delayed exit from cell cycle of progenitor cells and a delay of terminal differentiation, consistent with the ability of *PC3/Tis21* to inhibit cell cycle and promote differentiation [4], [12], [16].

**Figure 4:**
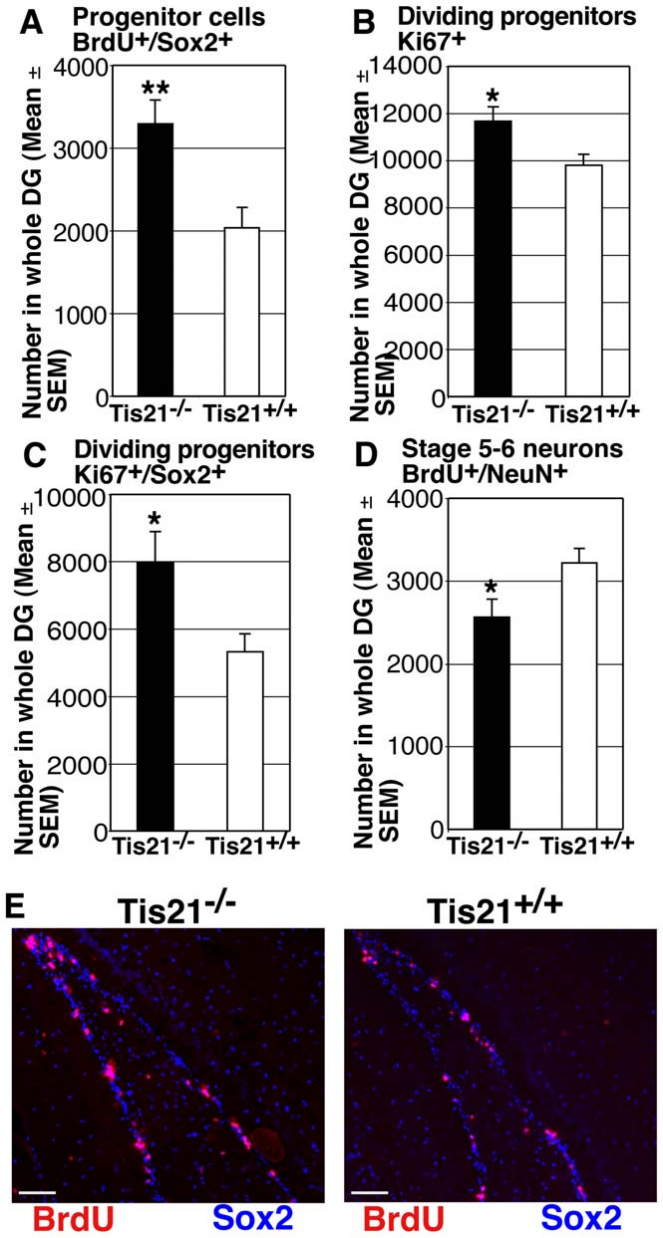
Increase of proliferating progenitor cells and decrease of terminally differentiated granule neurons in the immature dentate gyrus of *PC3/Tis21*-null mice at P14. (**A**) The whole population of progenitor cells entering in S-phase, labeled by a short pulse of BrdU (1hour) and by the progenitor cell marker Sox2 (BrdU/Sox2-positive), increased significantly in *PC3/Tis21*-null mice (1.6-fold). (**B**, **C**) The same significant increase is observed for the number of dividing progenitor cells, identified either as (**B**) Ki67-positive, or as (**C**) Ki67/Sox2-positive cells (1.5-fold). (**D**) The newly generated neurons (1 hour pulse of BrdU) decreased sharply (20%). Thus, also in the immature *PC3/Tis21*-null dentate gyrus prevails a condition of increased proliferation of progenitor cells and reduced differentiation of new neurons. Cell numbers are represented as mean ± SEM of the analysis of three animals per group. *, *p*<0.05 or **, *p*<0.01 vs. *PC3/Tis21* ^+/+^ dentate gyrus; Student's t test. (**E**) Representative confocal images of the dentate gyrus in P14 mice, showing cells double positive to BrdU/Sox2, in lower number in *PC3/Tis21*-null mice compared to WT. The merged images show that the new neurons incorporating BrdU (in red-pink) are localized quite exclusively in the proliferative subgranular zone and are virtually all positive also to the marker of proliferating progenitor cells Sox2. Scale bar, 75 µm.

Furthermore, we analyzed dentate gyrus progenitor cells and neurons at an earlier developmental stage (P14), when the population of proliferating progenitors is still expanding and begins to increase in the subgranular zone of dentate gyrus, while decreases in the tertiary matrix [61]. We used Sox2 to identify progenitor cells, where it is expressed during embryogenesis and in adult brain [50], [62], and Ki67 to label the whole population of dividing progenitor cells. We observed a significant increase of progenitor cells entering in S-phase, labeled by BrdU (a short pulse of 1 hour) and by Sox2 (BrdU^+^/Sox2^+^; 1.6-fold increase, *p* = 0.002; Figure 4A, E), and of the dividing progenitor cells labeled by Ki67, either alone (Ki67^+^; 18% increase, *p* = 0.01; Figure 4B) or with Sox2 (Ki67^+^/Sox2^+^; 1.5-fold increase, *p* = 0.02; Figure 4C). In contrast, the whole population of terminally differentiated dentate gyrus neurons decreased significantly (BrdU^+^/NeuN^+^; 20% decrease, *p* = 0.02; Figure 4D). Moreover, a significant decrease in the P14 dentate gyrus of *PC3/Tis21*-null mice was observed also in the total number of neurons expressing NeuroD1 (NeuroD1^+^, *PC3/Tis21* ^−/−^: 43065±3998; *PC3/Tis21* ^+/+^: 84285±2395; 3 mice analyzed per genotype; *p* = 0.0000, Student's t test).

Thus, the delay of terminal differentiation of neurons and the increase of progenitor cell proliferation observed in adult dentate gyrus was not restricted to adulthood, being detectable also at an earlier developmental stage.

**Figure 5:**
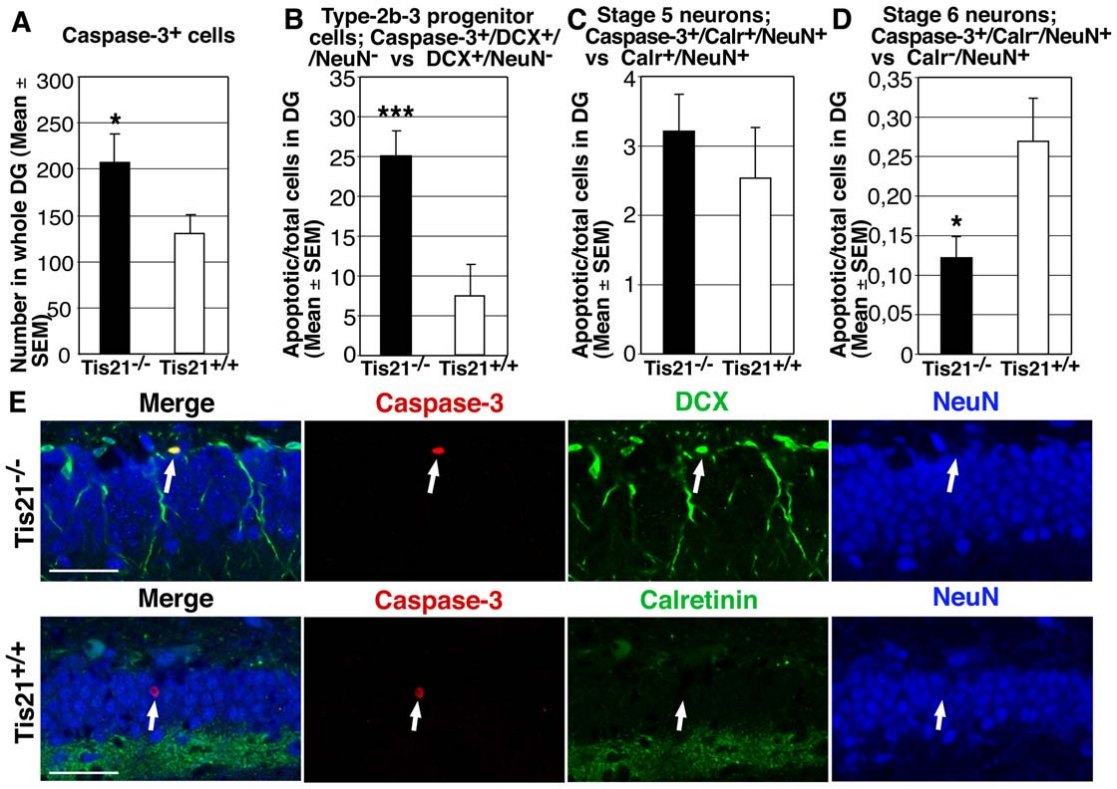
Cell survival analysis in *PC3/Tis21* ^−/−^ and *PC3/Tis21* ^+/+^ mice. (**A–D**) Cell survival was analyzed in the whole cell population of dentate gyrus of P60 normal and mutant mice and also in the different subpopulations of progenitor cells and neurons, using Caspase-3 as apoptotic marker. (**A**) A significant increase of the number of cells undergoing apoptosis was observed in all cells of dentate gyrus (1.6-fold increase) of *PC3/Tis21*-null mice, and more specifically in (**B**) type-2b and type-3 progenitor cells, measured as percentage of Caspase-3-DCX-positive and NeuN-negative cells in the population of DCX-positive and NeuN-negative cells (3.2-fold increase). (**C**) The percentage of undifferentiated apoptotic stage 5 neurons (Caspase-3/Calretinin/NeuN-positive in Calretinin/NeuN-positive) presented a not significant increase, while (**D**) a significant reduction was evident in the percentage of apoptotic stage 6 neurons in *PC3/Tis21*-null mice (Caspase-3/Calretinin-negative NeuN-positive in Calretinin-negative NeuN-positive; about 54% decrease). Thus, apoptosis was more frequent in progenitor cells type-2b and type-3 whose proliferation was increased. Cell numbers are represented as mean ± SEM of the analysis of three animals per group. *, *p*<0.05, or ***, *p*<0.001 vs. *PC3/Tis21* ^+/+^ dentate gyrus; Student's t test. (**E**) Confocal microscopy fields showing examples of apoptotic cells in the dentate gyrus of P60 mice, indicated by arrows, i.e., a type-2b/3 progenitor cell (Caspase-3/DCX-positive and/NeuN-negative) in *PC3/Tis21*-null mice, and a stage 6 neuron (Caspase-3/NeuN-positive and Calretinin-negative) in *PC3/Tis21* WT mice. Scale bar, 45 µm.

We also evaluated the survival of cells in the dentate gyrus, and observed that in *PC3/Tis21*-null mice the total number of apoptotic cells was greater than in WT, as detected by positivity to Caspase-3 (1.6-fold increase; *p* = 0.03; Figure 5A). Such increment, analyzed as percentage of cells undergoing apoptosis, was evident in the progenitor cell types whose proliferation increases significantly, i.e., type-2b and type-3 progenitor cells positive for DCX (Caspase-3^+^/DCX^+^/NeuN^−^; *p* = 0.0001; Figure 5B, E). On the other hand, we observed a not significant increase, of about 26%, of the percentage of stage 5 neurons undergoing apoptosis (Caspase-3^+^/Calret^+^/NeuN^+^; *p* = 0.41; Figure 5C). In stage 6 neurons, i.e., cells that had terminally differentiated, apoptosis was instead significantly less frequent in Ti21-null mice than in WT mice (Caspase3^+^/Calr^−^/NeuN^+^; 54% decrease; *p* = 0.01; Figure 5D, E). This suggests that the stage 6 neurons of *PC3/Tis21*-null mice, being in lower number than in WT (about 38% less than WT, Figure 2C), underwent a less competitive selection for survival [63].

Thus, an evident increase in the frequency of apoptosis occurred only in type-2b/type-3 proliferating progenitor cells, whose proliferation in *PC3/Tis21*-null mice was more pronounced than in control mice.

Furthermore, to test whether nonspecific changes occurred in mutant mice, we conducted a stereological analysis of the hippocampus volumes and cell numbers. No difference was observed between *PC3/Tis21*-null and WT mice at P60 and P14 in the volumes of the dentate gyrus or of the whole hippocampus, or in the total cell number of the dentate gyrus (see Table 1).

**Table 1:** Stereological analysis of hippocampal volumes and absolute cell numbers.

Parameter analyzed	Age	*PC3/Tis21* ^−/−^	*PC3/Tis21* ^+/+^	*p*-Value	n (mice)
Dentate gyrus volume (mm^3^)	P60	0.274±0.008	0.264±0.008	0.37	3
Dentate gyrus cell number	P60	413817±18499	403438±18011	0.69	3
Hippocampus volume (mm^3^)	P60	5.02±0.31	4.88±0.32	0.76	3
Dentate gyrus volume (mm^3^)	P14	0.186±0.010	0.186±0.005	0.96	3
Dentate gyrus cell number	P14	386598±27875	379426±10823	0.72	3

Statistical analysis was performed by Student's t test.

Additionally, we were interested in assessing whether the ablation of *PC3/Tis21* affected the maturation of the neurons of the subventricular zone, the other adult neurogenic brain region [64]. We observed that the number of new progenitor cells and neurons of the subventricular zone in P60 mice entering the S-phase, identified as BrdU^+^ cells after a BrdU treatment of 2 hours, increased significantly in Tis-21 null mutant (*PC3/Tis21* ^−/−^: 12872±605; *PC3/Tis21* ^+/+^: 10469±510; 3 mice analyzed per genotype; *p* = 0.002, Student's t test). In contrast, we observed a considerable reduction of the final number of differentiated neurons generated in the subventricular zone, as detected by measuring BrdU^+^/NeuN^+^ cells 4 weeks after their birth in the olfactory bulb, i.e., in their final migratory destination (*PC3/Tis21* ^−/−^: 32725±1346; *PC3/Tis21* ^+/+^: 38410±1269; 3 mice analyzed per genotype; *p* = 0.007, Student's t test). This indicated that *PC3/Tis21* was required for the control of proliferation as well as differentiation of adult progenitor cells in the subventricular zone, similarly to what observed in dentate gyrus.

### *PC3/Tis21*-Null Mice Show Normal Spatial Memory while Are Impaired in Contextual Fear Conditioning

As a further step, we sought to ascertain whether the delayed differentiation of dentate gyrus newborn neurons affected the hippocampus-dependent memory. Locomotor abilities and anxiety levels were preliminarily analyzed by submitting 2-month-old *PC3/Tis21*-null and WT mice to open field and plus maze tests [65], [66]. No statistically significant differences were found among the groups in both tasks (Figure S3). Then, spatial learning was tested in the Morris water maze, a task which is largely dependent on an intact hippocampus [41], [67].

**Figure 6:**
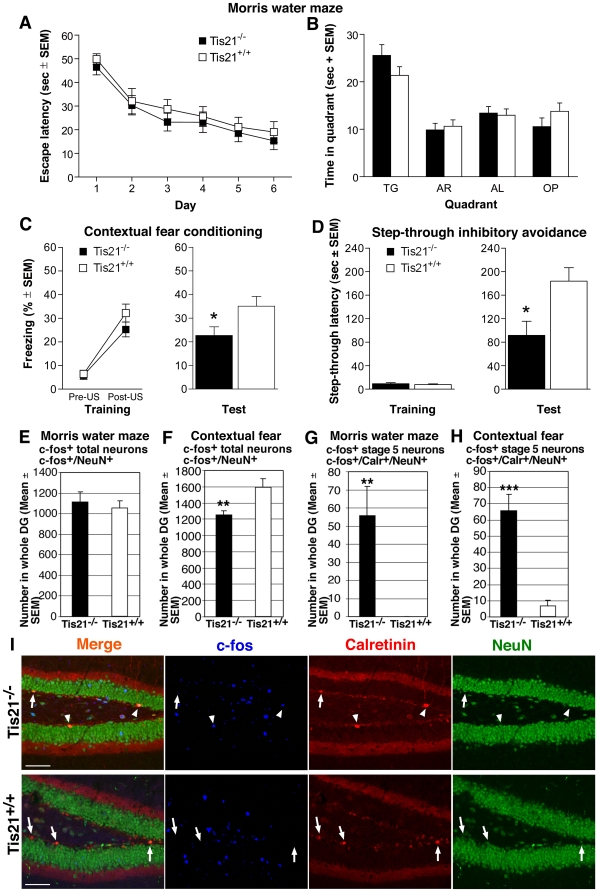
*PC3/Tis21* ^−/−^ mice show selective impairment of contextual memory and preferential recruitment of stage 5 neurons into dentate gyrus after both spatial and contextual behavioral task. (**A**) Escape latency (sec) throughout the Morris water maze behavioral test, to reach the hidden platform during the 6-day acquisition phase. (**B**) Time (sec) spent in the quadrants during the probe trial, when the hidden platform, located in the target quadrant (TG) during the acquisition phase, was removed. Both *PC3/Tis21* **^−/−^** and *PC3/Tis21* **^+/+^** mice spent a significantly greater amount of time in the TG quadrant, compared to the other quadrants. AR, adiacent right; AL, adiacent left; OP, opposite. (**C**) Contextual fear conditioning test: percentage of time spent in freezing behavior by *PC3/Tis21* ^−/−^ and *PC3/Tis21* ^+/+^ during both training (left) and test (right). During the training, no significant differences in the level of freezing between groups were observed both before (Pre-US) and after (Post-US) foot-shock administration. During the test, performed 24 h after training, *PC3/Tis21* ^−/−^ mice showed a significant reduction of freezing behavior compared to control mice. *, *p*<0.05, ANOVA. (**D**) Inhibitory avoidance test: mean latency to step-through into the dark compartment by *PC3/Tis21* ^−/−^ and *PC3/Tis21* ^+/+^ mice during both training (left) and test (right). During training, no significant differences were observed in the step-through latency between groups. During the test, performed 24 h after training, *PC3/Tis21* ^−/−^ mice showed a significant reduction of the step-through latency compared to that of control mice. *, *p*<0.05, ANOVA. (**E**) The activation into memory circuits of neurons within the whole dentate gyrus neuronal population (i.e., comprising stage 5 and 6) after the Morris water maze, measured as number of c-fos/NeuN-positive cells, was similar in *PC3/Tis21*-null and WT mice, while (**F**) after the contextual fear conditioning was significantly lower in *PC3/Tis21*-null mice. (**G**) The activation into memory circuits of stage 5 neurons, measured as number of c-fos/Calretinin/NeuN-positive cells, occurred quite exclusively in *PC3/Tis21*-null mice after the Morris water maze test and (**H**) also after the contextual fear conditioning test (being no c-fos/Calretinin/NeuN-positive neuron detectable in WT mice after the Morris water maze, or only few after the contextual fear conditioning test). This suggests a preferential recruitment in spatial memory networks of stage 5 neurons of *PC3/Tis21*-null mice. Analyses shown in (**E–H**) were performed in *PC3/Tis21* ^−/−^ and WT mice 1.5 hours after the end of behavioral test, expected to activate responsive neurons inducing c-fos expression. Cell numbers are represented as mean ± SEM of the analysis of three animals per group. **, *p*<0.01 or ***, *p*<0.001 vs. *PC3/Tis21* ^+/+^ dentate gyrus; Student's t test. (**I**) Representative confocal images showing stage 5 dentate gyrus neurons expressing c-fos, following behavioral training by the Morris water maze test, which are detectable only in *PC3/Tis21*-null mice (c-fos/Calretinin/NeuN-positive cells, in orange in the merged image, are indicated by arrowheads; single labeling is blue, red, green, respectively), or Calretinin/NeuN-positive c-fos-negative cells (indicated by arrows). Scale bar, 75 µm.

In this task, mice learn across daily sessions to find a hidden escape platform using extra-maze visual cues. *PC3/Tis21* ^−/−^ and control mice performed equally in the acquisition phase (Figure 6A). Statistical analysis (two-way ANOVA) showed a significant effect of training (F_(5,110)_ = 30.48; *p* = 0.0001), no significant effect of genotype (F_(1,22)_ = 0.55; *p* = 0.46) and no significant interaction between the two factors (F_(5,110)_ = 0.11; *p* = 0.98). In the probe test (Figure 6B), carried out 24 h after the last training trial, all mice spent a significantly greater amount of time in the target quadrant (F_(3,66)_ = 18.96; *p*<0.0001), irrespective of genotype (F_(1,22)_ = 2.96; *p* = 0.10).

We then investigated the effects of the ablation of *PC3/Tis21* in the contextual fear conditioning task, which involves mainly the hippocampus [68], [42]. In this task, immobility (freezing), a natural reaction elicited in mice by aversive stimuli, was recorded and considered as a measure of fear memory. Animals were trained in the conditioning chamber, where a foot-shock was delivered (unconditioned stimulus, US; see Materials and Methods). During the training (Figure 6C, left), no significant effect of the genotype was observed on the level of freezing (two-way ANOVA, F_(1,22)_ = 1.54; *p* = 0.23), and all mice reacted alike to the US (F_(1,22)_ = 89.37; *p*<0.0001). In the contextual test (Figure 6C, right), performed 24 h after training, *PC3/Tis21* ^−/−^ mice spent a significantly smaller amount of time in freezing behavior, compared to control mice (one-way ANOVA, F_(1,22)_ = 5.02; *p* = 0.03). Contextual memory was further investigated by submitting animals to an inhibitory avoidance task. In this task animals learn to avoid the dark compartment of an apparatus in which they receive an aversive stimulus [69]. During the training, before foot-shock administration (Figure 6D, left), mice showed no significant differences in the latencies to step-through into the dark compartment (F_(1,22)_ = 0.33; *p* = 0.57). In the test (Figure 6D, right) carried out 24 h after training, *PC3/Tis21* ^−/−^ mice showed significantly shorter step-through latencies compared to that of control mice (F_(1,22)_ = 7.65; *p* = 0.01).

### Stage 5 Neurons of *PC3/Tis21*-Null Mice Show Selective Incorporation into Dentate Gyrus Memory Networks after Behavioral Tasks

Mice tested for hippocampal memory were then analyzed for c-fos expression in dentate gyrus, to correlate their performance with the incorporation of neurons in spatial memory networks. In fact, c-fos is induced in dentate gyrus neurons that, following spatial memory training, are recruited into spatial memory circuits [34], [70].

1.5 hours after the end of the Morris water maze test, the activation of c-fos in the whole population of dentate gyrus neurons was equal in *PC3/Tis21*-null and WT mice, since no significant difference in number of c-fos^+^/NeuN^+^ neurons emerged (Figure 6E). In contrast, 1.5 hours after the end of the contextual fear conditioning, the whole population of c-fos^+^/NeuN^+^ neurons significantly decreased in *PC3/Tis21*-null mice, of about 21% (*p* = 0.006, Figure 6F). Representative confocal images are shown in Figure 6I.

Thus, after the Morris water maze, where no cognitive deficit was observed, an equal number of activated neurons was recruited from the whole neuron population into memory circuits of *PC3/Tis21*-null mice, despite the strong decrease of stage 6 terminally differentiated neurons.

As a further step, we evaluated whether the incompletely differentiated stage 5 neurons were integrated into memory circuits after each test, identifying them by c-fos, Calretinin and NeuN. Noteworthy, after the Morris water maze test and also after the fear conditioning test, we detected a significant number of activated stage 5 neurons in *PC3/Tis21*-null mice, while none, or very few, were present in WT mice (c-fos^+^/Calretinin^+^/NeuN^+^ cells; after Morris water maze: *p* = 0.002, Figure 6G; after contextual fear conditioning: *p* = 0.000002, Figure 6H).

As a whole, this indicated that: i) the extent of incorporation in memory circuits of neurons from the whole neuron population of dentate gyrus was closely correlated with the performance in the memory tests; ii) the incompletely differentiated stage 5 neurons were surprisingly activated in *PC3/Tis21*-null mice, after both memory tests. Since stage 5 neurons of 4 weeks of age are present also in WT mice (Figure 2B), this denotes a selective recruitment of this type of neurons in memory circuits of *PC3/Tis21*-null mice.

### *PC3* Is Recruited on the *Id3* Promoter and Negatively Regulates Id3 Expression

**Figure 7:**
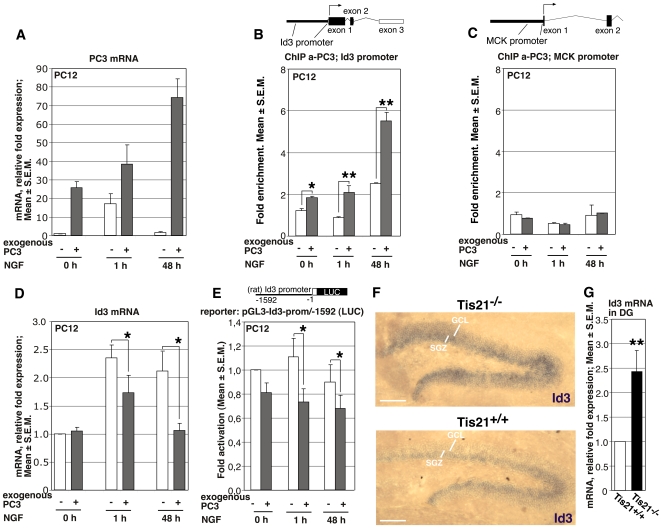
Binding of *PC3/Tis21* to the *Id3* promoter and corresponding decrease of *Id3* mRNA. (**A–D**) A PC12 cell line carrying an inducible *PC3* (rat) cDNA under control of the tet-off system (i.e., a tetracycline responsive element TRE-PC3, activated by a tetracycline-regulated transactivator, CMV-tTA, in the absence of doxycycline) was used to analyze the binding of *PC3* to the *Id3* promoter, by chromatin immunoprecipitation (ChIP). (**A**) PC12 cells without (white columns) or with expression of exogenous *PC3* (activated a week before by withdrawal of 2 µg/ml of doxycycline; grey columns) were exposed to NGF (100 ng/ml) for 1 and 48 hours. The levels of exogenous *PC3* increased considerably in the absence of NGF (t 0), and even more as the treatment with NGF proceeded. Endogenous *PC3* was also transiently i51duced by NGF after 1 hour, as expected (Bradbury et al., 1991). (**B**) ChIP analysis of *PC3* binding to the *Id3* promoter in PC12 cells, treated as shown in (**A**). A scheme above the graph describes the *Id3* gene and the promoter region analyzed, 700 nt 5′ to the transcription start. The amount of *Id3* promoter present in immunoprecipitates obtained using anti-*PC3* antibody is quantified by real time PCR and is expressed as fold enrichment (ratio of the percentage of the PC3-immunoprecipitated amount of *Id3* promoter detected in the input cell lysates to the percentage of the normal serum-immunoprecipitated amount detected in the input cell lysates). The binding of exogenous *PC3* (grey columns) to the *Id3* promoter increased in correlation with the increase of exogenous *PC3* mRNA levels (panel **A**). (**C**) No binding above background by *PC3* was observed on the negative control muscle creatin kinase (MCK) promoter. (**D**) *Id3* mRNA levels in differentiating PC12 cells (1 and 48 hours after NGF) decreased significantly, in correlation with the increase of binding of endogenous *PC3* to the *Id3* promoter. Mean ± SEM fold increases (mRNA) or fold enrichments (ChIP) are from three independent experiments performed in parallel for ChIP and mRNA analysis, using duplicate cultures of the same cells. *, *p*<0.05, or ** *p*<0.001 vs. the corresponding time point of PC12 cells without exogenous PC3, Student's t test. (**E**) *Id3* promoter activity in differentiating PC12 cells (1 and 48 hours after NGF) decreased significantly in correlation with the increase of binding of endogenous *PC3* to the *Id3* promoter. The *Id3* promoter construct comprised 1592 nt 5′ to the putative transcription start, placed upstream of a luciferase reporter (construct pGL3-*Id3*-prom/-1592). Luciferase activity is represented as mean ± SEM fold increase from four experiments. *, *p*<0.05 vs. the corresponding time point of PC12 cells without exogenous PC3, Student's t test. (**F**) The expression of *Id3* mRNA in dentate gyrus of P14 mice, as detected by in situ hybridization, increased considerably in *PC3/Tis21*-null mice, in the subgranular zone (SGZ) as well as in the granule cell layer (GCL). A representative image from one of three independent in situ experiments is shown. Scale bar, 80 µm. (**G**) The *Id3* mRNA levels in the dentate gyrus of P14 mice, measured by real time PCR after laser capture microdissection of the area, increased significantly in *PC3/Tis21*-null mice. Data are shown as mean ± SEM fold increase with respect to values obtained in WT, from three independent experiments. **, *p*<0.01 vs. WT mice, Student's t test (performed on data normalized to the endogenous controls but not yet relativized as fold-expression).

The marked delay of terminal differentiation of stage 6 neurons occurring in the absence of *PC3/Tis21* prompted us to investigate the underlying molecular mechanism. Given that *PC3/Tis21* is a transcriptional cofactor, which we have shown to be recruited to the promoter of *cyclin D1* and *RAR-β* [13], [14], we asked whether *PC3/Tis21* could bind also to the promoters of neural genes. To verify this possibility, we used a PC12 cell line - able to differentiate into sympathetic neurons upon addition of nerve growth factor (NGF) - expressing the tetracycline-regulatable tTA transactivator (PC12/tet-off). These cells were stably transfected with an inducible *PC3* gene (rat sequence) under control of a tetracycline responsive element. This system allowed us to induce high levels of exogenous *PC3* in the presence or in the absence of the differentiating stimulus, thus circumventing the limit presented by the narrow time-window of induction of endogenous *PC3* by NGF (in the range of 1 hour after treatment; Figure 7A). The induction of *PC3* mRNA was accompanied by induction of PC3 protein (data not shown). In these cells we analyzed, by chromatin immunoprecipitation (ChIP), the association of the PC3 protein to different neural gene promoters (namely, those of *NeuroD1*, *Hes1*, *Mash1*, *Ngn1*, *Msx1*, *Id1*, *Id3*; data not shown), observing that a significant association occurred only with the promoter of *Id3*, a gene that negatively regulates neural differentiation [71], [72], [73]. Indeed, we found that the amount of *Id3* promoter sequences recovered in the PC3 immunoprecipitates increased significantly above basal level both before and after NGF treatment, in correlation with the induction of exogenous *PC3* expression (Figure 7B; the increase of binding above the condition without induction of exogenous *PC3* was: 51% in the absence of NGF [t 0 hour], *p* = 0.01; 96% increase 1 hour after NGF addition, *p* = 0.007; 119% increase 48 hour after NGF addition, *p* = 0.002). In contrast, the recruitment of the PC3 protein to the muscle creatin kinase (MCK) promoter, which is inactive in PC12 cells and used here as negative control, was not above the background levels (Figure 7C). In parallel with the increased binding of PC3 to the *Id3* promoter, we observed a significant decrease of *Id3* mRNA levels upon induction of exogenous PC3, 1 and 48 hours after NGF treatment (Figure 7D; 26% decrease 1 hour after NGF addition, *p* = 0.01; 49% decrease 48 hours after NGF addition, *p* = 0.04). Next, we asked whether *PC3* could inhibit the *Id3* promoter activity. Therefore, we generated the construct pGL3-*Id3*-prom/-1592, which carries the *Id3* promoter region upstream of the luciferase reporter gene. This construct was transfected into PC12/tet-off cells, treated or not with NGF. *Id3* promoter activity was significantly reduced upon induction of exogenous PC3, 1 and 48 hours after NGF treatment, as compared to the activity in the absence of exogenous *PC3* (Figure 7E; 34% decrease 1 hour after NGF addition, *p* = 0.01; 25% decrease 48 hours after NGF addition, *p* = 0.03).

Taken together, these data clearly indicate that PC3 can bind the *Id3* promoter, and strongly suggest that it may inhibit the transcription of the anti-differentiative gene *Id3* through direct binding to its promoter.

We also sought to ascertain whether the regulation of *Id3* transcription by *PC3/Tis21* observed in differentiating PC12 cells occurred in vivo. To this aim, we analyzed by in situ hybridization the expression of *Id3* mRNA in the dentate gyrus - where it is normally expressed [74] - of WT or of *PC3/Tis21* ^−/−^ P14 mice, at a stage of high expansion of progenitor cells. We observed that in *PC3/Tis21* ^−/−^ mice, as compared to WT, not only *Id3* mRNA increased markedly in the proliferative subgranular zone, but also it was highly expressed ectopically in the granule cell layer where normally *Id3* is almost undetectable (Figure 7F). A significant increase of *Id3* mRNA in P14 *PC3/Tis21* ^−/−^ mice, with respect to WT mice, was observed also in the dentate gyrus microdissected by laser capture and analyzed by real time PCR (2.4-fold increase, *p* = 0.009; Figure 7G and Figure S4A-E). These data, confirming those acquired by in situ hybridization, are compatible with the results obtained in differentiating PC12 cells, and support the idea that *PC3/Tis21* negatively controls the expression of *Id3*, by inhibiting the *Id3* promoter activity.

**Figure 8:**
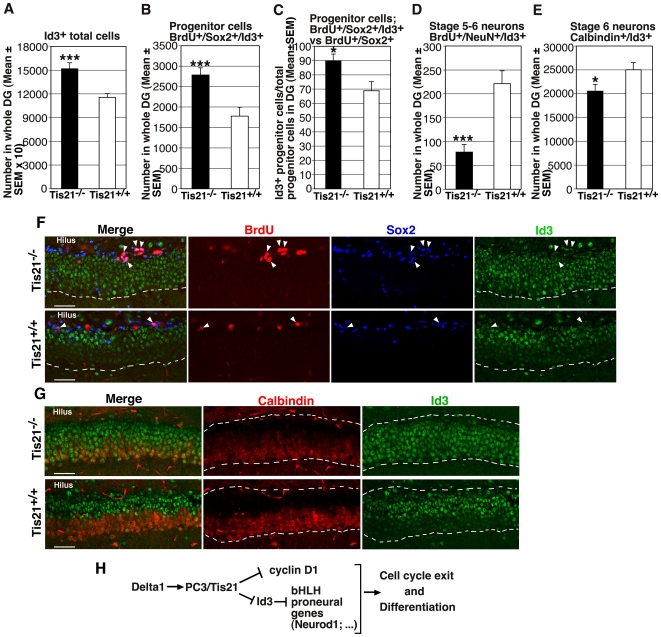
Increase of *Id3*-positive proliferating progenitor cells and decrease of *Id3*-positive differentiated neurons in the dentate gyrus of P14 *PC3/Tis21*-null mice. (**A**) In *PC3/Tis21*-null mice the total population of Id3-positive cells in the dentate gyrus and (**B**) the population of Id3-positive progenitor cells entering in S-phase, labeled by BrdU (1hour pulse) and by Sox2 (BrdU/Sox2/Id3-positive cells), increased significantly (1.31-fold and 1.56-fold, respectively). (**C**) Also the percentage of Id3-positive cells among the new proliferating progenitor cells (BrdU/Sox2/Id3-positive in BrdU/Sox2-positive) presented a significant increase. (**D**) In contrast, the new differentiating neurons expressing Id3 (BrdU/NeuN/Id3-positive neurons) decreased considerably, and (**E**) a significant decrease was observed also for the total population of terminally differentiated neurons co-expressing Id3 and Calbindin. Cell numbers are represented as mean ± SEM of the analysis of three animals per group. *, *p*<0.05 or ***, *p*<0.001 vs. *PC3/Tis21* ^+/+^ dentate gyrus; Student's t test. (**F, G**) Representative confocal images of the dentate gyrus in P14 mice; (**F**) The new proliferating progenitor cells expressing Id3, identified also by BrdU and Sox2 (BrdU/Sox2/Id3-positive, indicated by arrowheads; single labeling is red, blue, green, respectively) increase significantly in *PC3/Tis21*-null mice. It is also evident that the total number of Id3-positive cells increases greatly, with a localization expanded to the whole area of the granule cell layer, up to its outer third (delimited by the white broken line). Scale bar, 45 µm. (**G**) The whole population of terminally differentiated neurons, identified by Calbindin and co-expressing Id3, in *PC3/Tis21*-null mice is restricted toward the outer border of the dentate gyrus, in correspondence with the expansion to that area of the Id3-expressing cells. The white broken line delimits the inner and outer borders of dentate gyrus. Scale bar, 45 µm. (**H**) A model proposed for *PC3/Tis21* activity during neuronal differentiation (see Discussion). Delta1 is represented upstream of *PC3/Tis21*, whereas cyclin D1 downstream, in agreement with previous findings [78], [12].

We further sought to identify the cell populations of the dentate gyrus expressing the Id3 protein in *PC3/Tis21* ^−/−^ and WT mice, at P14. The total population of Id3-positive cells within the dentate gyrus of *PC3/Tis21* ^−/−^ mice increased considerably in number and in distribution, expanded over the whole granule cell layer up to the border with the molecular layer, consistently with the increase observed above for *Id3* mRNA expression (1.31-fold increase; Id3^+^, *p* = 0,0004; Figure 8A, F). New proliferating progenitor cells expressing *Id3*, identified by Sox2 and BrdU (1 hour pulse), were present in control mice but increased significantly in *PC3/Tis21*-null mice (1.56-fold increase; BrdU^+^/Sox2^+^/Id3^+^, *p* = 0.0008; Figure 8B, F). Moreover, the frequency (analyzed as percentage) of Id3-positive cells among the new proliferating progenitor cells increased significantly, suggesting a tight link between the increase of Id3 expression and the increase in number of progenitor cells observed in *PC3/Tis21*-null mice (BrdU^+^/Sox2^+^/Id3^+^ versus BrdU^+^/Sox2^+^; *p* = 0.016; Figure 8C). In contrast, we observed a dramatic decrease of the number of new differentiating neurons expressing Id3 (BrdU^+^/NeuN^+^/Id3^+^; 64% decrease, *p* = 0,00006; Figure 8D), and a significant decrease also of the whole population of terminally differentiated neurons co-expressing Id3 and Calbindin (Calbindin^+^/Id3^+^; 18% decrease, *p* = 0,04; Figure 8E). Notably, as shown in Figure 8G, in *PC3/Tis21-*null mice the population of Calbindin-positive cells, normally localized in the outer region of the dentate gyrus, decreased in number (Calbindin^+^, *PC3/Tis21* ^−/−^: 56056±5381; *PC3/Tis21* ^+/+^: 72740±1201; 3 mice analyzed per genotype; *p* = 0.016, Student's t test) and became more restricted toward the outer border of the dentate gyrus, correspondingly to the expansion of the area occupied by Id3-positive cells.

Altogether, this indicates that the increase of *Id3* expression is associated to the increase in number of proliferating progenitor cells and is also concomitant with the reduction of neurons attaining terminal differentiation observed in *PC3/Tis21*-null mice. This is compatible with the idea that the impaiment of terminal differentiation observed in the absence of *PC3/Tis21* is mediated by *Id3*.

## Discussion

This report provides the first evidence of a requirement of *PC3/Tis21* for neurogenesis. Previous studies have traced the expression of PC3/Tis21 in neural cells, exploiting a phenotypically wild type Tis21 GFP knock-in mouse, where the GFP sequence was fused to the second exon carrying the active domains of *PC3/Tis21* [8], [9], [15]. Here, in a *PC3/Tis21* knock out mouse model, we observe impairment of the last stage of maturation of hippocampal neurons, associated to deficit of contextual fear memory and to reduced incorporation of neurons into memory circuits. Key issues raised from these observations concern the underlying cellular and molecular mechanisms.

### Requirement of *PC3/Tis21* for Terminal Differentiation of Dentate Gyrus Neurons

The stage 5 hippocampal neuron is a post-mitotic cell type that in normal conditions is present only for a limited period after its birth, since it rapidly evolves into the stage 6 neuron [54]. In fact, in wild type mice we find that stage 5 neurons (BrdU+/Calr+/NeuN+) of 1–5 days of age are a small fraction of the terminally differentiated stage 6 neurons (BrdU+/Calr−/NeuN+), being less than one third. Moreover, at 28 days of age stage 5 neurons further decrease, their number becoming only 1/16 of stage 6 neurons. In mutant *PC3/Tis21* mice we observe that the number of stage 5 neurons of 1–5 days of age increases strikingly (about 2-fold), in comparison with normal mice, and remains higher also at 28 days of age, when the number of stage 5 neurons increases to represent 1/4 of stage 6 neurons. A similar increase is observed also for stage 5 neurons of 28 days of age still expressing DCX (i.e., BrdU+/DCX+/NeuN+).

Such major increase of stage 5 neurons, together with a significant reduction of terminally differentiated stage 6 neurons, clearly indicates that stage 5 neurons fail to mature into terminally differentiated neurons, and consequently accumulate. Therefore, stage 5 neurons of mutant mice are neurons whose final differentiation is almost indefinitely delayed by the absence of *PC3/Tis21* activity.

A similar delay of differentiation is observed also for the other adult neurogenic niche, the subventricular zone, in which neural cells do not attain terminal differentiation in the absence of *PC3/Tis21*, as the number of 28-day-old BrdU/NeuN-expressing neurons detected at P60 in the olfactory bulb, i.e., in their final migratory destination, decreases significantly.

As a whole, this suggests that *PC3/Tis21* is essential for the process of neuronal maturation.

### The Control of G1 Phase and of *Id3* Promoter Underlies the Role of *PC3/Tis21* in Differentiation

Moreover, the absence of *PC3/Tis21* led to an increase of proliferation, indicated by the greater number of progenitor cells in S-phase - labeled by a short 2 hours pulse of BrdU [29], [30] - and of the progenitor cells in active phases of cell cycle, identified by Ki67 [56], [75]. Remarkably, also the ratio between progenitor cells in S-phase (BrdU^+^) and cycling cells (Ki67^+^) increased, indicating that the cell cycle length was reduced. In fact, the ratio between BrdU- and Ki67-labeled cells accounts for G1 phase length because in mammalian cells the length of the S phase remains relatively constant while the length of G1 regulates proliferation [57], [58]. Furthermore, we did not observe any difference in the number of *PC3/Tis21*-null dentate gyrus cells in G2/M phase, as indicated by the phospho-histone H3 antigen. This reduction of the length of the G1 phase indicates that the control of the G1 phase is a physiological function of the gene *PC3/Tis21*, possibly exerted, according to our previous findings, by direct inhibition of the activity of the *cyclin D1* promoter [5], [13].

A primary effect of the reduction of G1 phase length occurring in the absence of *PC3/Tis21* may be a delay or inhibition of the neurogenic asymmetric division, to which the expression of *PC3/Tis21* is known to be associated in neuroblasts [6], [7], [1], [10]. Indeed, inhibition of the neurogenic asymmetric division can be the cellular mechanism at the origin of the increased proliferation observed in progenitor cells of mutant mice, which plausibly would be driven to preferentially divide symmetrically, thus generating a greater number of dividing cells.

On the other hand, the inhibition of the neurogenic asymmetric division alone may not account for the impairment of the terminal differentiation of stage 5 neurons into stage 6 neurons, dependent on *PC3/Tis21* ablation, given that stage 5 neurons are already post-mitotic. Without excluding the possibility that the action of *PC3/Tis21* on the cell cycle and cell division may have an influence on differentiation protracted also after mitosis, other mechanisms, likely involving the activity of proneural genes, should be implicated in the delayed differentiation of dentate gyrus neurons in Tis-21null mice. In this regard, we have observed the direct binding of *PC3/Tis21* to the promoter of *Id3*, which is an anti-differentiative gene [71], accompanied by down-regulation of Id3 mRNA expression and promoter activity, suggesting that PC3 may function as repressor of Id3. Accordingly, we observed an increase of *Id3* mRNA levels in the dentate gyrus in the absence of *PC3/Tis21*, accompanied by an increase of Id3-expressing proliferating progenitor cells and a parallel decrease of Id3-expressing differentiating neurons. *Id3* has a helix-loop-helix (HLH) dimerization domain, but lacks a DNA-binding domain and acts by sequestering E proteins, thus preventing their association to proneural basic HLH transcription factors, which are consequently inactivated [71], [72], [73]. One of these is *NeuroD1*, which is required for the maturation of hippocampal granule progenitor cells in differentiated neurons [55]. Consistently, we have also observed that *NeuroD1* expression decreases remarkably in dentate gyrus neurons of *PC3/Tis21*-null mice, compared to WT mice. Given that *PC3/Tis21* has been shown to bind histone deacetylases (HDACs) and to act as an inhibitory transcriptional co-factor [13], we can speculate that *PC3/Tis21* may impair the transcriptional activity of *Id3* by recruiting these inhibitory molecules to the *Id3* promoter, thereby facilitating the terminal differentiation of dentate gyrus neuron. Part of the transcriptional complex associated with the *Id3* promoter might also be Smad1 and Smad8 proteins, known to bind *PC3/Tis21* and to induce the levels of Id3 protein [28], [76], [77].

We have previously evidenced a control by *PC3/Tis21* on the activity of Math1 promoter in cerebellum [13], but here we present the first demonstration of direct molecular association to the promoter of a neural gene, i.e., *Id3*.

Remarkably, the expression of endogenous *PC3/Tis21*, present in type-2/type-3 progenitor cells and in stage 6 (Calbindin-positive) terminally differentiated neurons, but absent in stage 5 neurons [15], follows a pattern fully compatible with the alterations emerging after ablation of *PC3/Tis21* in the proliferation of progenitor cells and in the maturation of stage 5 neuron.

Altogether, our data suggest that *PC3/Tis21* is required to control the differentiation of hippocampal neurons through a dual regulation of cell cycle and of the activity of neural genes.

Thus, *PC3/Tis21* appears to be a non-redundant component of a pro-differentiative pathway, represented upstream by Delta1 [78] and, downstream, by *Id3* and its targets, i.e. the basic HLH proneural genes (Figure 8H).

### The Ablation of *PC3/Tis21* Causes a Deficit of Contextual Fear Memory and a Selective Recruitment in Memory Circuits of Stage 5 Undifferentiated Neurons

In previous studies adult neurogenesis was suppressed by different approaches, such as irradiation, genetic manipulation or cytostatic drugs [79]. A different paradigm, recently developed by us, targeted new neurons by inducing them to differentiate faster without altering their final number [16]. The majority of these approaches led to a significant memory impairment. In the present model, terminal differentiation of new neurons is prevented, while the proliferation rate of progenitor cells is increased.

This cellular phenotype is associated to different performances in memory tests, being a deficit evident in the contextual fear conditioning and inhibitory avoidance, but absent in the Morris water maze. A possible explanation is that fear-related tests, as one-time associative tasks, are more sensitive to the large decrease, up to 40%, of terminally differentiated stage 6 neurons 1- to 4-week-old, than the Morris water maze that, conversely, consists of repeated training trials. Consistently, the total number of c-fos activated neurons was equal in *PC3/Tis21*-null and WT mice only after the Morris water maze test, suggesting that a functional compensation of the decrease of terminally differentiated neurons occurred in memory circuits of *PC3/Tis21*-null mice during that test.

Notably, c-fos activation of stage 5 neurons (BrdU/Calretinin/NeuN-positive) occurred quite selectively in *PC3/Tis21*-null mice, after both Morris water maze and contextual fear conditioning tests. In fact, stage 5 neurons are present also in WT mice, which suggests that such preferential recruitment in memory circuits of stage 5 undifferentiated neurons may act as a homeostatic mechanism, compensating for the large reduction of stage 6 differentiated neurons in *PC3/Tis21*-null mice. However, since the undifferentiated stage 5 neurons in *PC3/Tis21*-null mice are equally recruited after both tests, it is unlikely that these neurons are responsible for the normal performance of mutant mice in the Morris water maze test. Nonetheless, we cannot exclude that, under certain learning conditions such as those of the Morris water maze test in *PC3/Tis21*-null mice, stage 5 neurons may contribute to recruit a larger number of neurons within memory circuits.

More generally, our data reveal how critical for spatial/contextual memory and recruitment in memory circuits of newborn neurons is the control of the neural differentiation process in dentate gyrus, not only at the stage of progenitor cells, as previously observed following an acceleration of their differentiation [16], but also for early post-mitotic neurons when their terminal differentiation is delayed, as observed here after genetic ablation of *PC3/Tis21*.

Moreover, given the physiological role in the control of proliferation and differentiation of developing neurons highlighted here for *PC3/Tis21*, and considering the neuroprotective role assigned to this gene in several systems [80], [81], [11], future studies should aim at defining whether *PC3/Tis21* plays a role in neurodegenerative diseases. In fact, a recent hypothesis implies the control of cell cycle in the onset of neurodegenerative diseases such as Alzheimer's disease, which impacts heavily on the hippocampal function [82].

## Supporting Information

Figure S1 Decrease of neurons expressing NeuroD1 in the dentate gyrus of PC3/Tis21-null mice. (A) In P60 PC3/Tis21-null mice the total number of NeuroD1-positive cells was significantly reduced, of about 15%. (B, C) Consistently, new neurons 1- to 5-day-old expressing NeuroD1, identified either as (B) BrdU/NeuroD1-positive, or as (C) BrdU/NeuroD1/NeuN-positive neurons, presented a significant decrease (of 22% and 35%, respectively). Immunoistochemical analysis was performed after five daily injections of BrdU. Cell numbers are represented as mean ± SEM of the analysis of three animals per group. *, p<0.05, or **, p<0.01 vs. PC3/Tis21+/+ dentate gyrus; Student's t test. (D) Representative confocal images showing dentate gyrus neurons of 1 to 5 days of age positive to BrdU/NeuroD1/NeuN (in orange in the merged image, indicated by arrowheads; single labeling is red, green, blue, respectively), which clearly decrease in PC3/Tis21-null mice. Scale bar, 40 µm.
(2.87 MB TIF)

Figure S2 Analysis of type-2ab progenitor cells through the Tbr2 marker. An analysis in the dentate gyrus of P60 PC3/Tis21-null and PC3/Tis21+/+ mice of the number of proliferating progenitors cells, using the type-2 population marker Tbr2, showed (A) no change within the whole type-2ab population (Ki67/Tbr2-positive, p = 0.88) or (B) in type-2a progenitor cells (Ki67/Tbr2-positive andDCX-negative, p = 0.71), and (C) a 25% increase of type-2b progenitor cells in PC3/Tis21-null mice (Ki67/Tbr2/DCX-positive, p = 0.60). Cell numbers are represented as mean ± SEM of the analysis of three animals per group. (D) Representative confocal images showing dentate gyrus neurons positive to Ki67/Tbr2/DCX (in bright yellow in the merged image, indicated by arrowheads; single labeling is green, red, blue, respectively; arrows indicate progenitor cells Ki67/Tbr2-positive and DCX-negative). Scale bar, 40 µm.
(5.71 MB TIF)

Figure S3 No differences between PC3/Tis21+/+ and PC3/Tis21−/− in basal behaviors and anxiety levels. (A-F) Basal behaviors and anxiety levels were evaluated in the open field and plus maze tests, respectively. (A-C) The open field test was carried out in a circular arena (60 cm of diameter) made in grey Plexiglas surrounded by walls (20 cm high). Animals were placed in the centre of the arena and allowed to explore it over a 8-min period. Elapsed the first 4 min, an object was inserted to the centre of the apparatus and mice were leaved into the arena for an additional 4-min period. Behaviors were videotaped and the time spent in locomotion (Loc), no locomotion (No loc), rearing (Rear), freezing (Freez) and contact with object (Cont), as well as the time spent in sectors (external, Ext; middle, Mid and internal, Int) were analyzed by using the Observer software (Noldus Information Technology, Costerweg, NL). No significant differences were observed between PC3/Tis21+/+ and PC3/Tis21−/− mice (A) in all behaviors recorded, (B) in the time spent in the sectors, and (C) in mean velocity. (D-F) The plus maze was carried out in a grey Plexiglas elevated maze with four arms 30 cm long and 5 cm wide extending from a central starting platform. Two opposite arms were enclosed by grey walls (15 cm high) and two arms were open. Animals were placed in the centre of the apparatus and allowed to explore it for 5 min. Behaviors were videotaped and the time spent in central platform and in both closed and open arms were analyzed. (D) No significant differences in the time spent in both closed and open arms (Closed, Open) and in the central platform (Central) between PC3/Tis21+/+ and PC3/Tis21−/− mice were observed. No significant differences in the time spent in locomotion (Loc) and no locomotion (no loc) (E), as well as in the distance moved (F) during the test were also observed.
(0.17 MB PDF)

Figure S4 Laser capture microdissection of the dentate gyrus in P14 PC3/Tis21-null mice. (A-E) Representative images outlining the computer-assisted procedure of laser capture microdissection of the dentate gyrus, from cresyl violet-stained histological slides obtained from P14 PC3/Tis21-null or WT mice. (A, B) 4x magnification images of the whole hippocampus before and after removal of the laser dissected dentate gyrus region. Scale bar, 200 µm (C, D, E) 20x magnification images of the dentate gyrus before and after the laser cut, and after removal of the area. Scale bar, 200 µm.
(0.29 MB PDF)
