# Supplementary figures and images for: Impaired Terminal Differentiation of Hippocampal Granule Neurons and Defective Contextual Memory in PC3/Tis21 Knockout Mice
Source: PLoS One. 2009 Dec 17;4(12):e8339. doi: 10.1371/journal.pone.0008339 (PMC2791842; doi:10.1371/journal.pone.0008339)

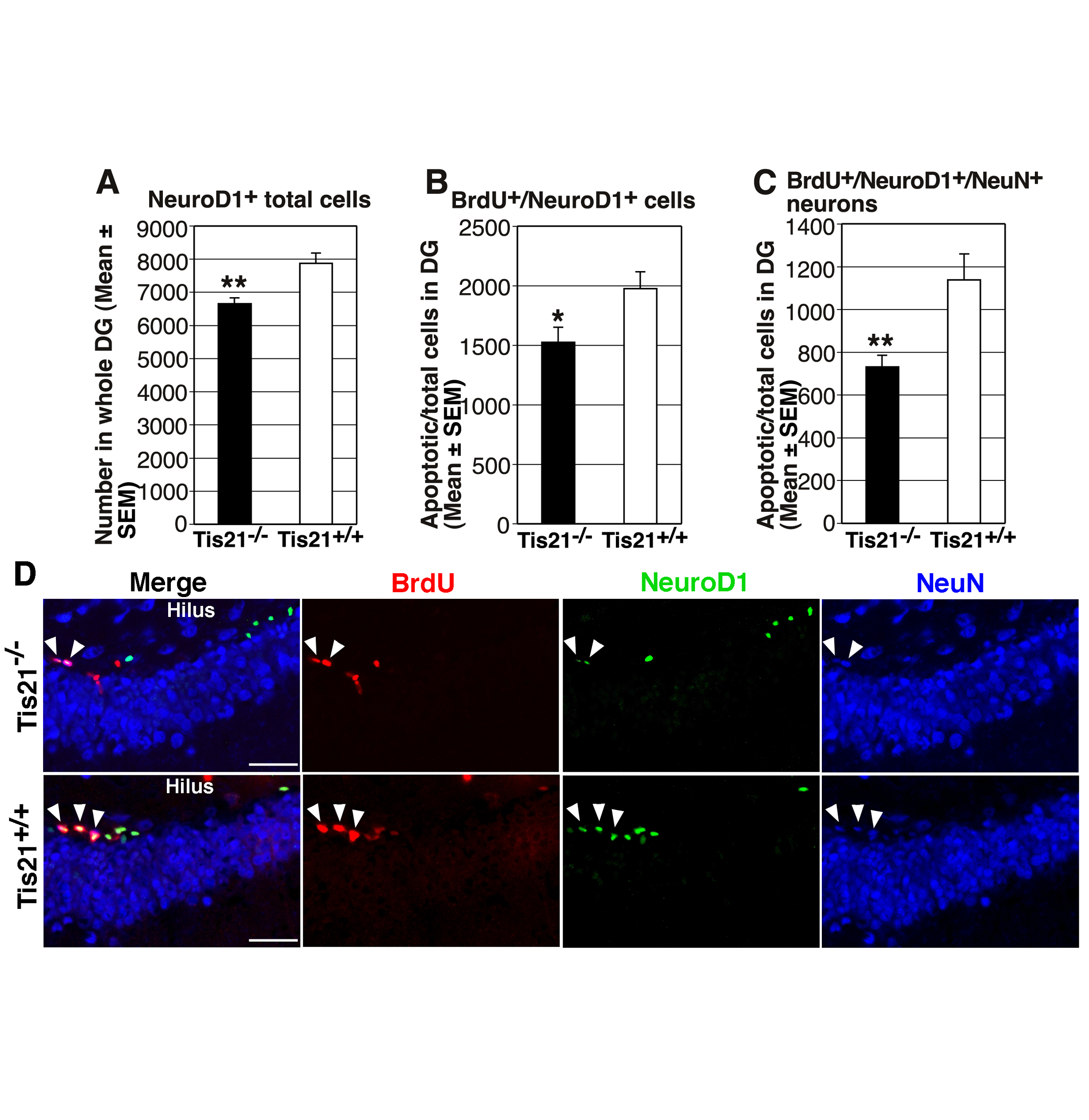

Supplement: Figure S1 — Decrease of neurons expressing NeuroD1 in the dentate gyrus of PC3/Tis21-null mice. (A) In P60 PC3/Tis21-null mice the total number of NeuroD1-positive cells was significantly reduced, of about 15%. (B, C) Consistently, new neurons 1- to 5-day-old expressing NeuroD1, identified either as (B) BrdU/NeuroD1-positive, or as (C) BrdU/NeuroD1/NeuN-positive neurons, presented a significant decrease (of 22% and 35%, respectively). Immunoistochemical analysis was performed after five daily injections of BrdU. Cell numbers are represented as mean ± SEM of the analysis of three animals per group. *, p<0.05, or **, p<0.01 vs. PC3/Tis21+/+ dentate gyrus; Student's t test. (D) Representative confocal images showing dentate gyrus neurons of 1 to 5 days of age positive to BrdU/NeuroD1/NeuN (in orange in the merged image, indicated by arrowheads; single labeling is red, green, blue, respectively), which clearly decrease in PC3/Tis21-null mice. Scale bar, 40 µm. (2.87 MB TIF) [file pone.0008339.s001.tif]

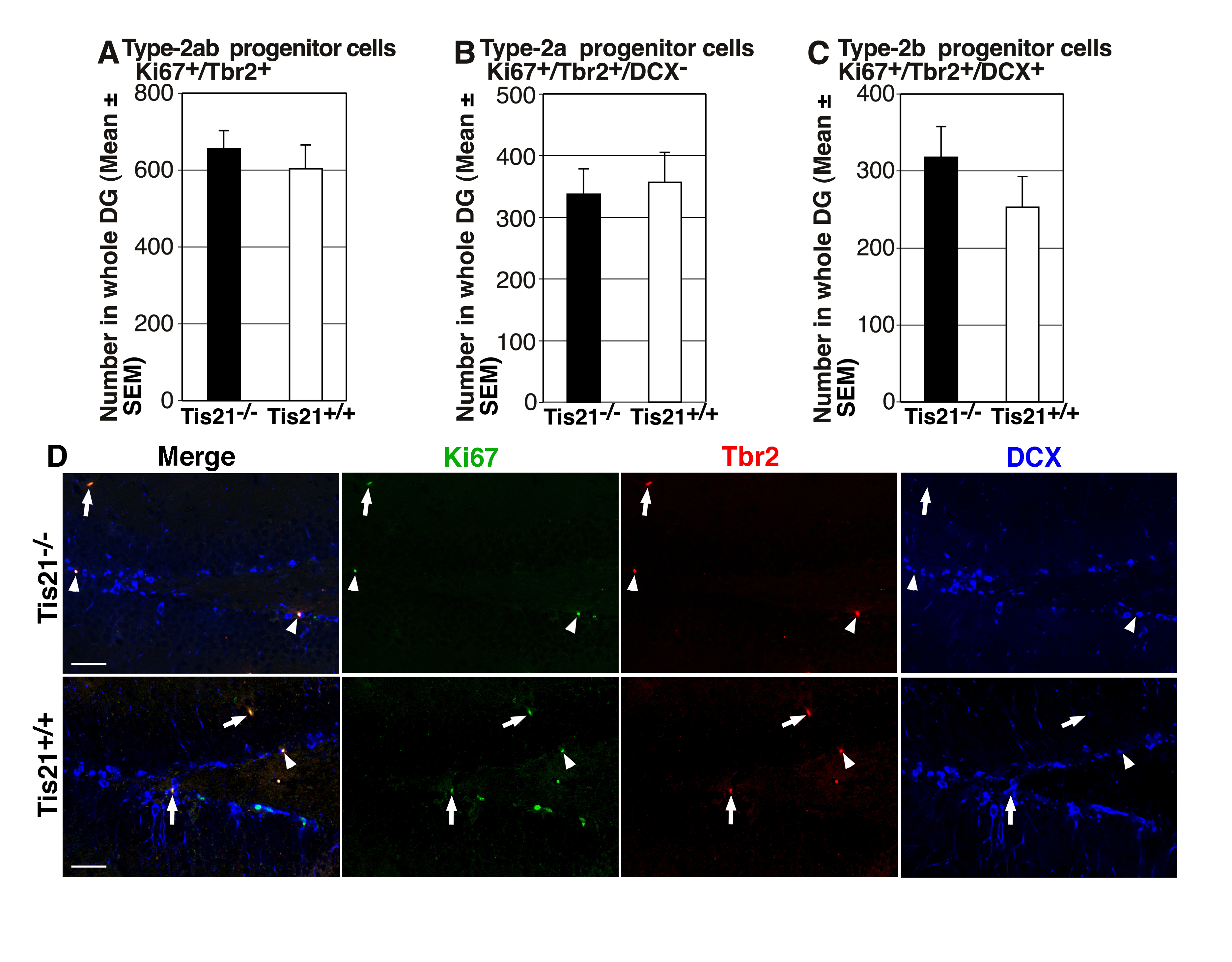

Supplement: Figure S2 — Analysis of type-2ab progenitor cells through the Tbr2 marker. An analysis in the dentate gyrus of P60 PC3/Tis21-null and PC3/Tis21+/+ mice of the number of proliferating progenitors cells, using the type-2 population marker Tbr2, showed (A) no change within the whole type-2ab population (Ki67/Tbr2-positive, p = 0.88) or (B) in type-2a progenitor cells (Ki67/Tbr2-positive andDCX-negative, p = 0.71), and (C) a 25% increase of type-2b progenitor cells in PC3/Tis21-null mice (Ki67/Tbr2/DCX-positive, p = 0.60). Cell numbers are represented as mean ± SEM of the analysis of three animals per group. (D) Representative confocal images showing dentate gyrus neurons positive to Ki67/Tbr2/DCX (in bright yellow in the merged image, indicated by arrowheads; single labeling is green, red, blue, respectively; arrows indicate progenitor cells Ki67/Tbr2-positive and DCX-negative). Scale bar, 40 µm. (5.71 MB TIF) [file pone.0008339.s002.tif]

# Open Field

**A**

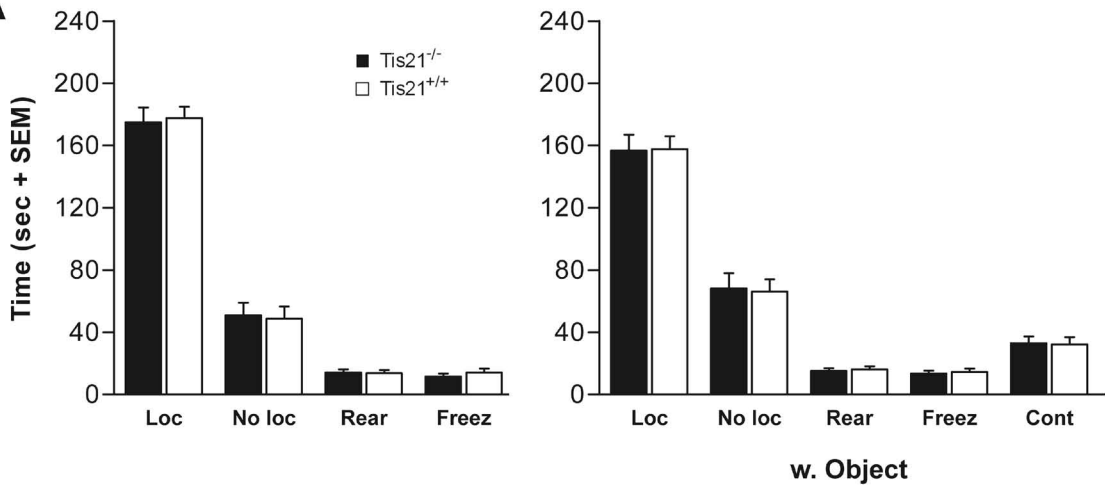

**B**

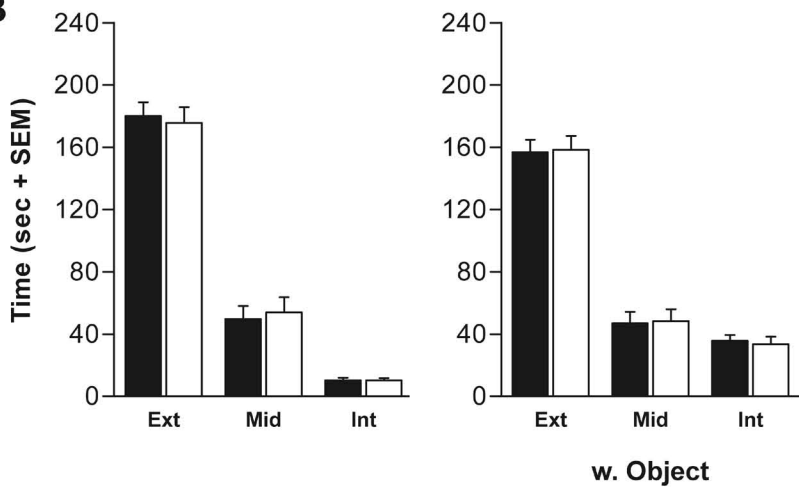

**C**

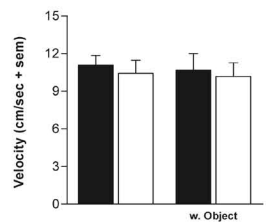

# Plus Maze

**D**

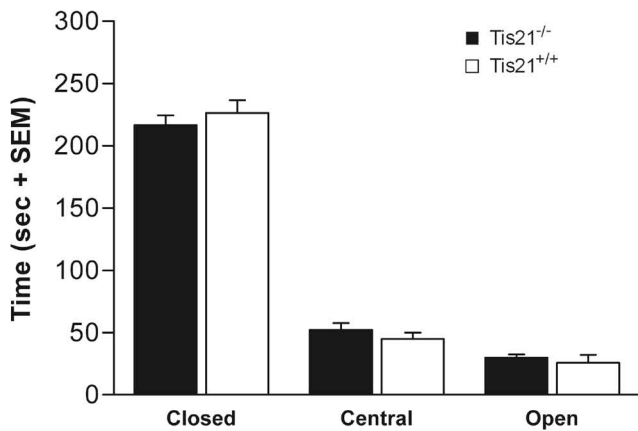

**E**

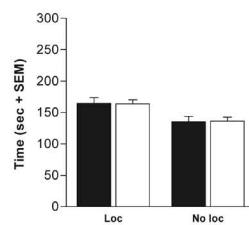

**F**

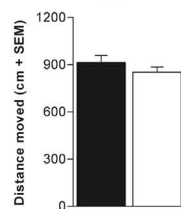

Supplement: Figure S3 — No differences between PC3/Tis21+/+ and PC3/Tis21−/− in basal behaviors and anxiety levels. (A-F) Basal behaviors and anxiety levels were evaluated in the open field and plus maze tests, respectively. (A-C) The open field test was carried out in a circular arena (60 cm of diameter) made in grey Plexiglas surrounded by walls (20 cm high). Animals were placed in the centre of the arena and allowed to explore it over a 8-min period. Elapsed the first 4 min, an object was inserted to the centre of the apparatus and mice were leaved into the arena for an additional 4-min period. Behaviors were videotaped and the time spent in locomotion (Loc), no locomotion (No loc), rearing (Rear), freezing (Freez) and contact with object (Cont), as well as the time spent in sectors (external, Ext; middle, Mid and internal, Int) were analyzed by using the Observer software (Noldus Information Technology, Costerweg, NL). No significant differences were observed between PC3/Tis21+/+ and PC3/Tis21−/− mice (A) in all behaviors recorded, (B) in the time spent in the sectors, and (C) in mean velocity. (D-F) The plus maze was carried out in a grey Plexiglas elevated maze with four arms 30 cm long and 5 cm wide extending from a central starting platform. Two opposite arms were enclosed by grey walls (15 cm high) and two arms were open. Animals were placed in the centre of the apparatus and allowed to explore it for 5 min. Behaviors were videotaped and the time spent in central platform and in both closed and open arms were analyzed. (D) No significant differences in the time spent in both closed and open arms (Closed, Open) and in the central platform (Central) between PC3/Tis21+/+ and PC3/Tis21−/− mice were observed. No significant differences in the time spent in locomotion (Loc) and no locomotion (no loc) (E), as well as in the distance moved (F) during the test were also observed. (0.17 MB PDF) [file pone.0008339.s003.pdf]

**A**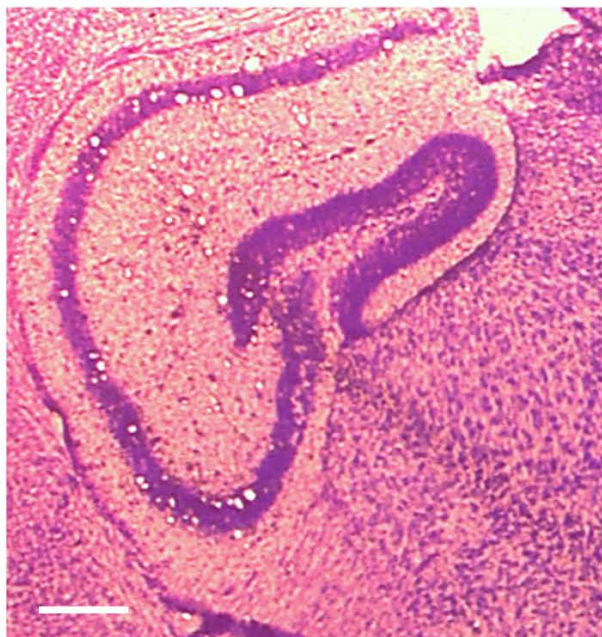**B**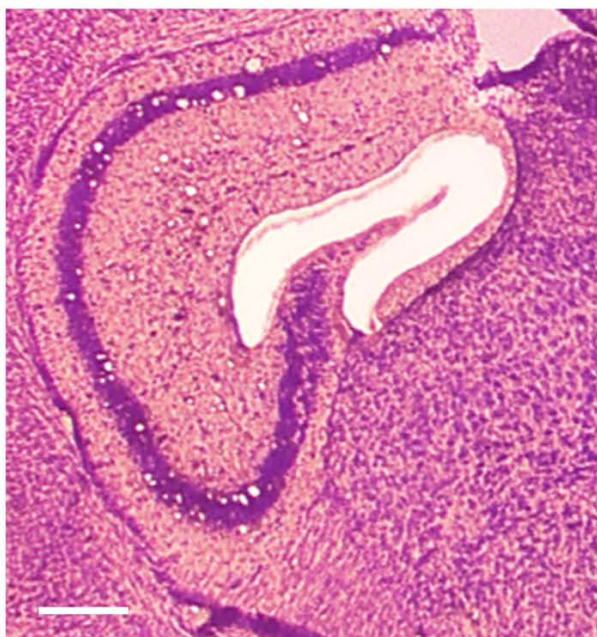**C**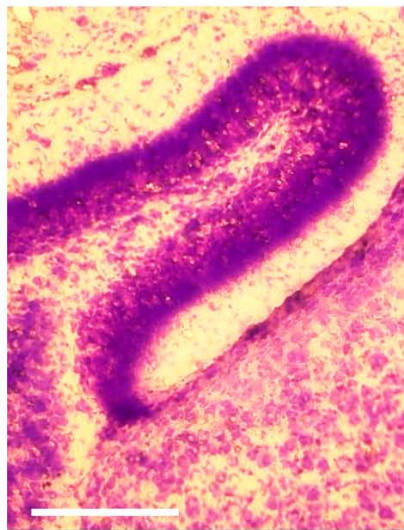**D**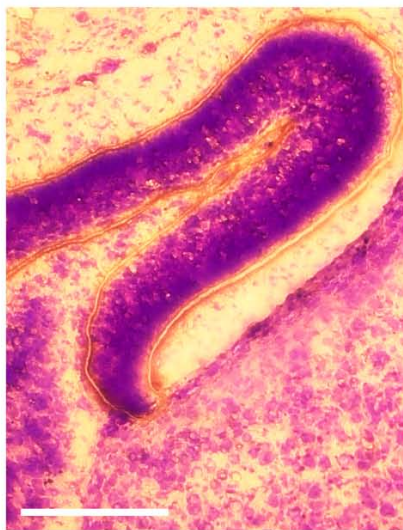**E**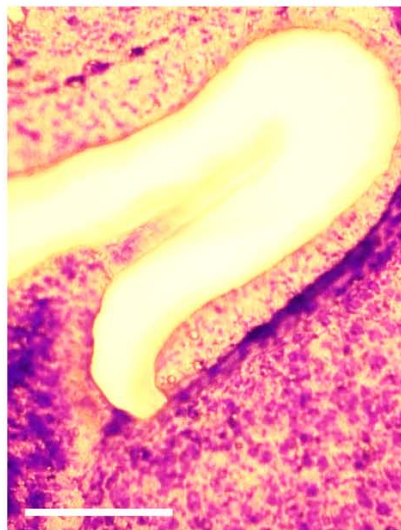

Figure S4

Supplement: Figure S4 — Laser capture microdissection of the dentate gyrus in P14 PC3/Tis21-null mice. (A-E) Representative images outlining the computer-assisted procedure of laser capture microdissection of the dentate gyrus, from cresyl violet-stained histological slides obtained from P14 PC3/Tis21-null or WT mice. (A, B) 4x magnification images of the whole hippocampus before and after removal of the laser dissected dentate gyrus region. Scale bar, 200 µm (C, D, E) 20x magnification images of the dentate gyrus before and after the laser cut, and after removal of the area. Scale bar, 200 µm. (0.29 MB PDF) [file pone.0008339.s004.pdf]
